# Repeated Parental Singing During Kangaroo Care Improved Neural Processing of Speech Sound Changes in Preterm Infants at Term Age

**DOI:** 10.3389/fnins.2021.686027

**Published:** 2021-09-03

**Authors:** Kaisamari Kostilainen, Eino Partanen, Kaija Mikkola, Valtteri Wikström, Satu Pakarinen, Vineta Fellman, Minna Huotilainen

**Affiliations:** ^1^Cognitive Brain Research Unit, Department of Psychology and Logopedics, Faculty of Medicine, University of Helsinki, Helsinki, Finland; ^2^New Children’s Hospital, Pediatric Research Center, Neonatology, Department of Pediatrics, University of Helsinki and Helsinki University Hospital, Helsinki, Finland; ^3^Pediatrics, Department of Clinical Sciences, Lund University, Lund, Sweden; ^4^Children’s Hospital, University of Helsinki, Helsinki, Finland; ^5^CICERO Learning Network, Faculty of Educational Sciences, University of Helsinki, Helsinki, Finland

**Keywords:** auditory event-related potential, auditory processing, infant-directed singing, mismatch response, preterm birth, sound discrimination

## Abstract

Preterm birth carries a risk for adverse neurodevelopment. Cognitive dysfunctions, such as language disorders may manifest as atypical sound discrimination already in early infancy. As infant-directed singing has been shown to enhance language acquisition in infants, we examined whether parental singing during skin-to-skin care (kangaroo care) improves speech sound discrimination in preterm infants. Forty-five preterm infants born between 26 and 33 gestational weeks (GW) and their parents participated in this cluster-randomized controlled trial (ClinicalTrials ID IRB00003181SK). In both groups, parents conducted kangaroo care during 33–40 GW. In the singing intervention group (*n* = 24), a certified music therapist guided parents to sing or hum during daily kangaroo care. In the control group (*n* = 21), parents conducted standard kangaroo care and were not instructed to use their voices. Parents in both groups reported the duration of daily intervention. Auditory event-related potentials were recorded with electroencephalogram at term age using a multi-feature paradigm consisting of phonetic and emotional speech sound changes and a one-deviant oddball paradigm with pure tones. In the multi-feature paradigm, prominent mismatch responses (MMR) were elicited to the emotional sounds and many of the phonetic deviants in the singing intervention group and in the control group to some of the emotional and phonetic deviants. A group difference was found as the MMRs were larger in the singing intervention group, mainly due to larger MMRs being elicited to the emotional sounds, especially in females. The overall duration of the singing intervention (range 15–63 days) was positively associated with the MMR amplitudes for both phonetic and emotional stimuli in both sexes, unlike the daily singing time (range 8–120 min/day). In the oddball paradigm, MMRs for the non-speech sounds were elicited in both groups and no group differences nor connections between the singing time and the response amplitudes were found. These results imply that repeated parental singing during kangaroo care improved auditory discrimination of phonetic and emotional speech sounds in preterm infants at term age. Regular singing routines can be recommended for parents to promote the development of the auditory system and auditory processing of speech sounds in preterm infants.

## Introduction

Preterm birth interrupts intrauterine fetal development and increases the risk of neurodevelopmental abnormalities (Jarjour, 2015). The adverse developmental outcomes in preterm infants vary from major neurological deficits, such as cognitive, visual, and hearing impairments to more common minor cognitive dysfunctions of attention and language (Anderson et al., 2003; Jarjour, 2015). Minor cognitive deficits at later ages have been associated with atypical auditory processing already in infancy (Mikkola et al., 2007; Jansson-Verkasalo et al., 2010; Hövel et al., 2014).

Preterm birth affects the development of the auditory system. The uteral auditory environment consisting of predictable rhythmic low-frequency sounds and familiar speech sounds changes to the extrauterine auditory environment in the neonatal intensive care unit (NICU). In addition to changes in other sensory experiences, infants are exposed to sudden and unpredictable high-frequency alarm and monitor sounds, which are not dampened by the uterus and amniotic fluid. Exposure to these stimuli not only causes physiological stress but can also disturb the infant’s neurological development (Rand and Lahav, 2013). Along with experiencing unnatural auditory stimuli, preterm infants are at risk of parental voice deprivation during their stay in the NICU (Coppola and Cassibba, 2010; Caskey et al., 2011; Rand and Lahav, 2013). These factors together with brain immaturity, have been connected to the later development delays in this population (McMahon et al., 2012; Lahav and Skoe, 2014).

Early auditory sensory experience affects brain development. After preterm birth, the brain continues to develop and grow outside the sheltering uterus. The change of the sensory environment occurs at an active cortical gyration phase, and during this time, the brain is especially fragile and sensitive to the surrounding sensory stimuli. Auditory deprivation or exposure to unstructured and loud sounds may disturb development. For example, Lu et al. (2008) found auditory deprivation during the fetal period to alter the development of the rat auditory cortex, while Chang and Merzenich (2003) showed that rats exposed to continuous noise during the fetal period had abnormal cortical structures after birth. On the contrary, developmentally relevant auditory stimuli have been shown to promote brain development. For example, Webb et al. (2015) demonstrated in their randomized study, including 40 extremely preterm infants, that listening to recorded mother’s voice and heartbeat sounds positively influenced brain plasticity. In their results, infants exposed to the recordings during the first month of postnatal life had significantly larger auditory cortices at one month of age when compared to those infants that were not exposed to the recordings. Recorded and live maternal sounds, speech and singing have also supported the development of preterm infants by improving their feeding (Krueger et al., 2010; Chorna et al., 2014), behavioral and cardiorespiratory stability (see Filippa et al., 2017), cardiorespiratory regulation (Doheny et al., 2012), and neurobehavioral development (Picciolini et al., 2014).

Infant-directed (ID) singing, which has been defined as musical nurturing of the child by regulating their arousal and mood and engaging infant attention effectively (Trehub, 2019), can offer parents of hospitalized infants a way to use their voices and interact sensitively taking into consideration their infant’s medical condition (O’Gorman, 2006). Studies made with typically developing infants show that usually, the songs for infants by the caregivers include more emotional content, such as a higher pitch, slower tempo, and longer inter-phase pauses (Trainor et al., 1997; Nakata and Trehub, 2011). Together with clear rhythm and predictability, these prosodic features are believed to attract infant attention (Trainor, 1996), enhance the detection of phonemes and words (Ma et al., 2011; Graf Estes and Hurley, 2013), and promote language acquisition by making word segmentation easier (Thiessen et al., 2005; Adriaans and Swingley, 2017; Goswami, 2019).

Taken together, preterm birth exposes the fetus to an environment that vastly differs from the normal developmental conditions and auditory stimulation that infants at the same gestation experience in the uterus. This drastic change in the auditory environment can alter the development of the auditory system and increase the risk for abnormal neurodevelopment. There is evidence that developmentally appropriate auditory stimuli, such as maternal speech and singing, can positively affect preterm infant development (Filippa et al., 2017). It is also known that the prosodic features in ID singing promote language acquisition in typically developing infants. However, the effects of parental singing have not been directly investigated on the development of auditory processing and discrimination of speech sounds in preterm infants.

Auditory perception and processing in infants can be studied with auditory event-related potentials (AERP), the time-locked events of the electroencephalogram (EEG). The mismatch negativity (MMN), a component of AERP, is a neural response elicited in the temporal and frontal cortical areas of the brain in adults, when an infrequent deviant stimulus in a sound sequence is found to be different from the frequently presented standard stimulus (Alho et al., 1990; Näätänen et al., 1978, 2010). MMN is elicited already at birth, and in infancy, it is often referred to as the mismatch response (MMR), because the polarity of the responses can vary and both positively and negatively displaced responses occur due to maturational factors (Leppänen et al., 2004; He et al., 2007) and varying stimulation paradigms (Háden et al., 2009; Cheng et al., 2015).

We studied whether creating a sensory multimodal experience with daily parental singing during standard skin-to-skin care (kangaroo care) would beneficially impact the auditory processing in preterm infants at term age. We hypothesized that adding parental singing to kangaroo care would improve the maturation of the auditory system and enhance the change-detection processing of speech sounds as indexed by the MMRs. We furthermore assumed that the amount of parental singing during the intervention would lead to larger MMRs, indicating more enhanced auditory change-detection processing in these infants.

## Materials and Methods

### Study Design

The data were collected from the Finnish cohort of the longitudinal two-country Singing Kangaroo randomized controlled trial (ClinicalTrials ID IRB00003181SK). The preterm infants were born in Helsinki University Central Hospital. After intensive care, the infants were transferred to either the neonatal ward L2 in Jorvi Hospital or neonatal ward LV37 in Kätilöopisto Maternity Hospital for further neonatal care. To avoid the contamination of the groups, cluster-randomization was implemented in these two neonatal wards, and families were allocated either to a singing intervention group or a control group, depending on to which ward they were transferred. At the halfway of the recruitment period, the hospitals’ assignments were switched so that the intervention and the control groups would include families from both neonatal wards.

**FIGURE 1:**
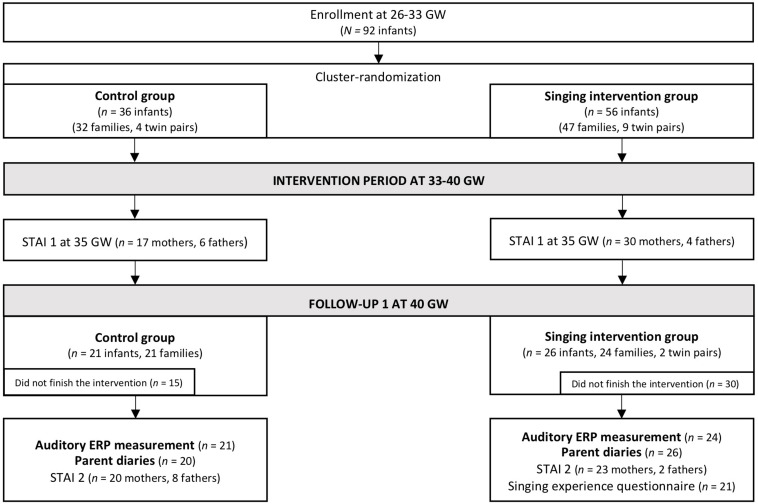
Flowchart of the Finnish cohort of the Singing Kangaroo study (adapted from Kostilainen et al., 2021). The data used in the current study are highlighted in bold (the results of the STAI and questionnaire data are reported in Kostilainen et al., 2021).

The inclusion criterion was that the infants had been born between 26 and 33 GW to Finnish-speaking families. The exclusion criteria were cerebral hemorrhage stages III–IV, congenital central nervous system abnormalities, and the need for ventilator support. However, infants treated with continuous positive airway pressure (CPAP) were eligible. Before discharge from the neonatal ward, the infants’ hearing was tested with an otoacoustic emission screening (MADSEN AccuScreen, Budapest, Hungary). The ethical statement for the study was obtained from the Ethics Committee of Hospital District of Helsinki (Ethics Committee for gynecology and obstetrics, pediatrics, and psychiatry 65/13/03/03/2012) and research permission was awarded by the Helsinki University Central Hospital. The parents received both written and oral information of the study protocol and gave thereafter their written informed consent. The flowchart of the Singing Kangaroo study protocol is presented in Figure 1.

### Intervention

In both groups, parents were instructed to conduct kangaroo care, preferably at least 1.5–2 h a day. In the Finnish neonatal wards, kangaroo care (e.g., Moore et al., 2014; Boundy et al., 2016) is a standard care practice, and parents are encouraged to conduct it daily as soon as the infant’s condition is stable enough. The parents in both groups started the intervention in the hospital as early as possible, and preferably at 33 GW at the latest. They were instructed to continue with the daily sessions independently at the hospital and after discharge until their infant reached 40 GW. These late GWs were chosen for the intervention because during these weeks there is an accelerated increase in cortical gyration, synaptic density, and myelination, that make the auditory cortex capable of receiving external stimuli. We assumed that during this developmental period, it might be possible to increase the effect of auditory stimulation on the development of the auditory cortex.

Before starting the intervention, a certified music therapist (KK) instructed the parents in the *singing intervention group* to sing or hum in an infant-directed way during the daily kangaroo care. The music therapist gave approximately 10–15-min verbal instructions about how to create a soothing and relaxing sound environment with parental singing and how to avoid overstimulation. Thus, lullabies with simple melodies sang with a slow tempo, and a low volume level were recommended (O’Gorman, 2006) for preterm infants with lower GWs and also when putting infants to sleep. The singing intervention period was several weeks long, and the developmental needs of the infants were to change during these postpartum weeks. Therefore, the parents were guided by the possibility of singing more active children’s songs when their infants started to reach full-term age and search for more active interaction while awake for more extended periods. To support parents in conducting the singing intervention, the music therapist offered a self-made song booklet, including Finnish lullabies and children’s songs. The parents were also encouraged to sing songs that were meaningful to them, e.g., songs from their childhood with emotional relevance (Loewy et al., 2013; Loewy, 2015). In contrast, parents in the *control group* were not specifically encouraged to sing or instructed to use their voices, but to carry out standard kangaroo care. Hence, they performed daily kangaroo care as a standard practice until their infants reached 40 GW.

### Participants

Overall, 92 preterm infants (singing intervention group, *n* = 56; control group, *n* = 36) and their parents initially participated in the Finnish cohort of the Singing Kangaroo study. The parents of 30 preterm infants in the singing intervention group and 15 preterm infants in the control group did not complete the intervention period. Hence, the infants of these parents did not participate in the AERP measurement after the intervention period. Furthermore, two preterm infants from the intervention group did not participate in the AERP measurement even though the parents completed the intervention period. From the remaining 24 infants in the singing intervention group, the data of two infants were omitted from the primary analysis due to parents not singing during the intervention. In turn, from the remaining 21 infants in the control group (partially the same as in Kostilainen et al., 2020), the data of one infant were omitted from the primary analysis due to parents listening to recorded music repeatedly during the intervention. Regardless, the data of these three families were added to the filtered data when conducting an additional sensitivity analysis according to the intention-to-treat (ITT) principle recommended for randomized controlled trials.

Moreover, in the control group, the data of one infant were omitted from further analysis due to incomplete data files and one infant due to gestational age at birth exceeding the limit set for inclusion criteria. For the final analysis, the data of 22 preterm infants in the singing intervention group and 18 preterm infants in the control group were included. The participant information of the preterm infants is reported in Table 1. The groups did not statistically significantly differ regarding the birth characteristics, except for the sex distribution.

**TABLE 1:** The participant information of the preterm infants (mean and range).

	Singing intervention group	Control group
Preterm infants, *n*	22	18
Male (%)	17 (77%)	7 (39%)
Gestational weeks at birth	30.6 (26.7–33.3)	30.3 (27.1–33.3)
Weight (g)	1529.8 (900–2800)	1342.9 (925–1950)
Height (cm)	40.6 (35–48)	39.3 (35.4–45)
SGA^a^, *n*	2	4
Umbilical cord arterial pH	7.26 (6.98–7.34)	7.24 (7.11–7.38, *n* = 15)
Apgar scores at 5 min^b^	6.6 (1–10, *n* = 20)	7 (4–9, *n* = 17)
Gestational weeks at AERP measurement	41.2 (38.9–44.1)	40.5 (38.3–42.7)

*^*a*^Born as small for gestational age, birth weight of less than −2 standard deviations for the age.^*b*^Newborn health assessment on a scale 1–10, assessed 5 min postnatally (Breathing effort, heart rate, muscle tone, reflexes, and skin color).*

### Data Collection and Analysis

#### Parent Diary

Parents registered the duration of daily intervention in diaries. In the singing intervention group, parents reported the duration of kangaroo care (min) and singing (min). Parents in the control group reported the duration of kangaroo care (min) and wrote a description of the auditory environment in each care situation (e.g., silence, talking, and singing). The parents returned the parent diary when they brought their infant to the AERP measurement at term age. In the singing intervention group, all 22 parents returned the parent diaries. In the control group, one family did not return the diary, resulting in 17 parent diaries for analysis.

#### AERP Measurement

At term age, an AERP measurement was performed with EEG using two different sound paradigms: a multi-feature paradigm with phonetic and emotional speech sound changes and a one-deviant oddball paradigm with pure tones. These two different sound paradigms were chosen because they enabled the assessment of both speech sound and non-speech sound discrimination. In the measurement, the multi-feature paradigm with speech sounds was presented first. This order of presentation was chosen since the multi-feature paradigm offers a more constant, sleep-enhancing sound pattern combined with the oddball paradigm.

##### Multi-Feature Paradigm

**FIGURE 2:**
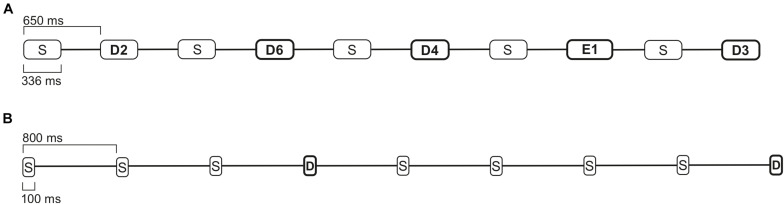
The paradigm examples of 7 s intervals. **(A)** Multi-feature paradigm with standard (S), phonetic deviant (D), and emotional stimuli (E), with a 650 ms SOA. **(B)** A one-deviant oddball paradigm with standard (S) and deviant stimuli (D), with an 800 ms SOA.

The multi-feature paradigm used in this study was originally developed by Pakarinen et al. (2014), and also used in our previous studies with a reduced number of deviant sounds (Kostilainen et al., 2018, 2020). The paradigm consisted of a 336 ms Finnish bi-syllabic pseudoword /ta-ta/ as a standard sound (with 46% probability, 700 in each block). The deviants included nine deviants (six phonetic and three emotional stimuli). The six phonetic deviant stimuli were: *vowel duration change* /ta-ta:/ (with 11% probability, 175 in each block); *vowel change* /ta-to/ (with 11% probability, 175 in each block); *intensity changes* ± *6 dB* (with 5% probability each, 77 each in one block); and *frequency changes* ± 25.5 Hz (*D3/G3*) (with 6% probability each, 98 each in one block). The three emotional deviant stimuli *happy*, *sad*, and *angry*, were natural utterances of the standard sound /ta-ta/. Each of them rarely occurred in the sound stream (with a 3% probability each, 42 each in one block). The sounds in the paradigm were presented with a 650 ms stimulus onset asynchrony (SOA), with every other sound either being a standard sound or a deviant (Table 2a and Figure 2A).

**TABLE 2:** Detailed paradigm information.

(a) The multi-feature paradigm with standard, deviant, and emotional stimuli (Pakarinen et al., 2014, same as in Kostilainen et al., 2018, 2020).					

**Stimulus**	**Utterance**	**Total duration (ms)**	**1st syllable (ms)**	**2nd syllable (ms)**	**Deviance information**

**Standard**	/ta-ta/	336	168	168	Frequencies: 1st syllable 175 Hz and 2nd syllable 168.5 Hz Intensity: 2nd syllable –2.5 dB
**Emotional stimulus**					
Happy	/ta-ta:/	388	125	263	Frequencies: 276 and 177 Hz Intensities: +1 and –2 dB
Sad	/ta-ta:/	436	218	218	Frequencies: 196 and 163 Hz Intensities: +3 and –6 dB
Angry	/ta-ta/	337	125	212	Frequencies: 276 and 260 Hz Intensities: –1 and –2 dB
**Deviant stimulus**					
Vowel duration	/ta-ta:/	400	168	232	Frequencies: 168 and 162 Hz Intensity difference of Std from the 1st syllable: –2 dB
Vowel change	/ta-to/	336	168	168	Frequencies: 175 and 168.5 Hz Intensity difference from Std: <1 dB
Intensity change	/ta-ta/	336	168	168	Intensity: 2nd syllable ±6 dB (50% each): perceived as loudness changes
Frequency change	/ta-ta/	336	168	168	Frequencies: 2nd syllable ±25.5 Hz (50% each): perceived as pitch changes

(b) Oddball paradigm with standard and deviant stimuli (same as in Kostilainen et al., 2020).							

**Stimulus**	**Pure tone**	**Total duration (ms)**	**Deviance information**		

Standard	1000 Hz	100			
Deviant	1100 Hz	100	Pitch change		

The speech sounds in the multi-feature paradigm were presented by a female Finnish speaker. In the phonetic deviants, *vowel duration change* /ta-ta:/ and *vowel change* /ta-to/, the main changes occurred in the second syllable. As the sounds were naturally uttered, they differed slightly from the standard sound already in the first syllable in some features. The phonetic deviants, *intensity changes* ± *6 dB*, and *frequency changes* ± 25.5 Hz (D3/G3), in turn, were modified digitally from the standard sound. In these deviants, the change occurred only in the second syllable compared to the standard stimulus. The emotional deviants (*happy, sad, and angry*) were prosodically exaggerated natural utterances and differed from the standard sound already from the onset of the first syllable (see Supplementary Material for an audio clip example of the multi-feature paradigm and Supplementary Figure 1 for spectrograms of the standard, deviant and emotional stimuli). To ensure that the emotional stimuli were perceived as intended, adult listeners (*n* = 5) rated the sounds using five basic emotions listing (see Supplementary Table 1).

##### Oddball Paradigm

The one-deviant oddball paradigm with pure tones consisted of a 100 ms standard sound of 1000 Hz (with 80% probability, 800 in one block) and a deviant sound of 1100 Hz (20% probability, 200 in one block). In the sound sequence, the stimuli were pseudo-randomly presented with an 800 ms SOA. Between the deviant sounds, always at least one standard stimulus was presented, and no consecutive deviants were played (see Table 2b and Figure 2B; an audio clip example of oddball paradigm is presented in the Supplementary Material).

### Data Recording and Processing

The EEG was recorded on average at 40.9 GW (range 38.3–44.1 GW) by a registered research nurse while the infants were mainly in active or quiet sleep. The sound paradigms were played through Genelec 8010 loudspeakers placed about 50 cm behind the head of the infant using Presentation 17.2 (Neurobehavioral Systems Ltd., Berkeley, CA, United States). Two consecutive stimulation blocks of the multi-feature paradigm (duration of one stimulation block approximately 17 min) and one stimulation block of the oddball paradigm (duration approximately 14 min) were played. The International 10–20 System electrode location was used, and EEG was recorded from the electrodes F3, F4, C3, Cz, C4, T3, T4, P3, and P4 (Low cutoff DC, high cutoff 100 Hz, sampling rate 250 Hz) with Neuroscan SynAmps 2 (Compumedics USA Inc., United States).

The EEG data were processed with MATLAB R2018a (The MathWorks Inc., Natick, MA, United States) and MATLAB toolbox EEGLAB 13.6.5b (Delorme and Makeig, 2004). Data visualization and quantification were done using CBRUplugin2.0b (Tommi Makkonen, Cognitive Brain Research Unit, University of Helsinki, Finland), which is an in-house toolbox running on EEGLAB. The left mastoid electrode was used for referencing the EEG online. A high-pass filter of 1 Hz and a low-pass filter of 20 Hz were used, and the mean value of the left and right mastoid electrodes were used for re-referencing the EEG offline. The epochs were set to start 100 ms prior to the stimulus onset and to end 650 ms after the stimulus onset. The mean value of the period of 100–0 ms before the stimulus onset was used as a baseline by subtracting it from the signal at 0–650 ms. Any epochs with signal values exceeding the limit of ±150 μV were considered to contain artifacts and were omitted from the analysis. The number of accepted epochs for each stimulus in both paradigms are presented in Table 3.

**TABLE 3:** The accepted epochs in both paradigms.

(a) The accepted epochs for the standard, deviants, and emotional sounds in the multi-feature paradigm (two stimulation blocks combined).										

**Stimulus**	**Std**	**Dev1**	**Dev2**	**Dev3**	**Dev4**	**Dev5**	**Dev6**	**Happy**	**Sad**	**Angry**

Total epochs	1400	350	350	154	154	196	196	84	84	84
*Singing intervention group*										
Min	951	230	240	107	108	138	123	62	60	63
Max	1392	348	349	154	154	195	196	83	84	84
Mean	1338.4	334.6	335	148	147.8	187.4	187.4	79.8	80.8	80.8
mean (%)	95.6	95.6	95.7	96.1	96	95.6	95.6	95	96.2	96.2
*Control group*										
Min	1063	264	262	116	118	153	147	67	63	59
Max	1396	349	349	154	154	195	196	84	83	84
Mean	1307.5	327.2	326.1	142.8	144.3	182.6	183.5	79.3	77.9	79
mean (%)	93.4	93.5	93.2	92.7	93.7	93.2	93.6	94.4	92.7	94.1

(b) The accepted epochs for the standard and deviant sound in the oddball paradigm.										

**Stimulus**	**Std**	**Dev**								

Total epochs	800	200								
*Singing intervention group*										
Min	601	157								
Max	799	200								
Mean	773.9	193.9								
mean (%)	96.7	97								
*Control group*										
Min	536	133								
Max	794	200								
Mean	762.8	190.5								
mean (%)	95.4	95.3								

*Dev1: vowel duration; Dev2: vowel change; Dev3: intensity +6 dB; Dev4: intensity −6 dB; Dev5: frequency +25.5 Hz; Dev6: frequency −25.5 Hz.*

### Statistical Analysis

The statistical analyses were carried out with SPSS 26 (IBM Corporation, NY, United States), and the alpha level was set to 0.05. To set the latency windows of interest, the brain responses for the standard and each stimulus were compared using point-by-point *t*-tests. As the MMR is elicited in the frontocentral electrodes, the averaged values of the frontal electrodes F3, F4, and central electrodes C3 and C4 combined were used for the analysis (Supplementary Figures 2, 3). According to the results of both this point-by-point *t*-test analysis and our previous infant AERP study (Kostilainen et al., 2020), the latency windows between 200–300, 400–500, and 550–650 ms were set for both paradigms. The grand averages of both groups were created by averaging each participant’s accepted epochs for each electrode and stimulus within the chosen latency windows and by collecting the averaged values of the standard-subtracted brain responses within these latency windows for further analysis.

As the recruitment for this study was implemented in two hospital settings using a cluster-randomization method, the birth characteristics of the infants were compared between the two hospitals, and no differences regarding the participant information in the neonatal wards were found (birth characteristics according to the recruiting hospital reported in Supplementary Table 2). The mean values, standard deviations, and range were calculated from the parent diaries regarding the intervention duration. To determine whether an MMR was elicited, one-sample *t*-tests were used to compare if the standard-subtracted mean values differed significantly from 0 μV. The results of the *t*-tests (*t*- and *p*-values, means, standard deviations, and 95% confidence intervals) are reported fully in Supplementary Table 3. Repeated-measures ANOVA (rmANOVA) was used to examine the between-group condition and within-group comparisons in each of the latency windows. The between-group factors were Group (singing intervention group, control group) and Sex (female, male). Sex was added as a variable since it is known to affect outcomes in preterm infants (see O’Driscoll et al., 2018). The within-group factors included Stimulus (multi-feature paradigm: nine; six phonetic deviants and three emotional sounds) and Electrode (both paradigms: F3, F4, C3, and C4). In the multi-feature paradigm, only the emotional sounds were analyzed at the early latency window of 200–300 ms since, in the phonetic deviants, the actual deviant change occurred not until the second syllable.

Spearman’s rank-order correlation test showed no relationship between the daily singing time (min) and the magnitude of the MMRs in any of the latency windows in both paradigms in the singing intervention group. However, the overall singing intervention duration (days) correlated positively with several phonetic and emotional deviants in all three examined latency windows in the multi-feature paradigm. To avoid multiple testing and type 1 error, a repeated-measures analysis of covariance (rmANCOVA) was carried out for the final analysis while controlling for the overall singing intervention duration in days. According to our previous study results (Kostilainen et al., 2020), there was no reason to assume that the birth weight or gestational age at birth would have affected the magnitude of the MMRs and were not added as covariates. In the analyses, Greenhouse-Geisser correction was used when the assumption of sphericity was not met, and *post hoc* tests were conducted using Bonferroni correction (only corrected values and uncorrected degrees of freedom reported). Effect sizes are reported using partial eta squared (η^2^). The main and interaction effects of the Electrode factor were excluded from the main results and are partly reported in the Supplementary Material and Supplementary Table 4.

Finally, to follow the ITT principle recommended for randomized controlled trials, the between-group comparisons were rerun with all the available data included in the analysis. Hence, in this sensitivity analysis, the data from those two families in the singing intervention group that did not sing and data from one family in the control group that repeatedly listened to music during the intervention were added to the filtered data. The results of the ITT analysis are reported separately in the Supplementary Material and in Supplementary Table 5. Briefly summarized, the rmANOVA results showed the same main and interaction effects and did not considerably differ from the analysis done for the filtered set.

## Results

### Parent Diary

In the singing intervention group, kangaroo care was implemented on average 132 min per day (range 26–304, SD 52), and parents sang, on average, 42 min per day (range 8–120, SD 36). The mean intervention duration for kangaroo care was 42 days (range 25–68, SD 11), of which parents sang on average for 36 days (range 15–63, SD 12). In the control group, parents carried out kangaroo care on average 144 min per day (range 72–354, SD 63), and the mean overall duration of the intervention was 48 days (range 9–77, SD 17). According to the parent diaries, the auditory environment during the kangaroo care in the control group did not include singing. No statistically significant differences between the groups in kangaroo care duration both per day (min) and intervention duration (days) were found.

### AERP Measurement

#### Multi-Feature Paradigm

In all three studied latency windows, distinct MMRs were elicited to the emotional stimuli and many of the phonetic deviants in the singing intervention group. In the control group, MMRs were evoked to some of the emotional stimuli and phonetic deviants. The results of the one-sample *t*-test are reported in detail in Supplementary Table 3. In the first latency window 200–300 ms, the rmANOVA showed a main effect of Group [*F*(1, 36) = 7.077, *p* = 0.012, η^2^ = 0.164], as the singing group infants’ responses were larger than those of control group infants (singing intervention group 5.731 μV; control group 2.801 μV). A main effect of Stimulus [*F*(2, 35) = 5.987, *p* = 0.004, η^2^ = 0.143] was found: the responses for emotional stimulus *happy* were statistically larger than the responses for emotional stimuli *sad, p* = 0.027, and *angry, p* = 0.009 (*happy* 5.672 μV; *sad* 3.729 μV; *angry* 3.397 μV). Furthermore, an interaction effect of Group and Sex [*F*(1, 36) = 10.059, *p* = 0.003, η^2^ = 0.218] was revealed, as the responses of the female infants were larger than those of the male infants in the singing intervention group, *p* = 0.003 (singing intervention females 8.487 μV; singing intervention males 2.974 μV).

**FIGURE 3:**
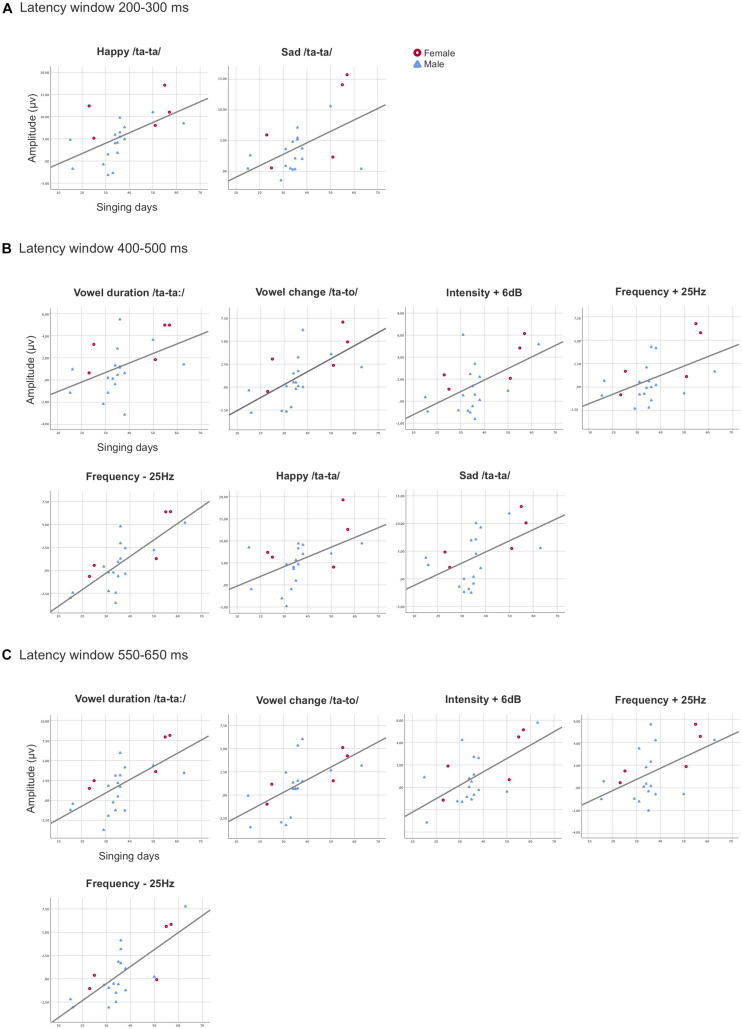
Scatterplots demonstrating the statistically significant associations found between the overall singing days and the MMR amplitudes in the singing intervention group for the averaged values of the electrodes F3, F4, C3, and C4 combined in the latency windows 200–300 ms **(A)**, 400–500 ms **(B)**, and 550–650 ms **(C)**. The X-axis represents the duration of overall singing days (range from 15 to 63 days) and the y-axis represents the amplitude (μV) of the responses.

In the latency window 200–300 ms, the rmANCOVA, with the overall singing days controlled, showed a main effect of Sex, [*F*(1, 19) = 8.815, *p* = 0.008, η^2^ = 0.317], as the female preterm infants had statistically larger MMRs when compared to the male preterm infants (adjusted means: singing intervention females 7.839 μV, singing intervention males 3.165 μV). An interaction effect of Stimulus and Singing days was found [*F*(2, 18) = 3.834, *p* = 0.030, η^2^ = 0.168], as the longer singing intervention was related to larger MMRs for the emotional sounds *happy* and *sad* (adjusted means: *happy* 6.993 μV, *sad* 4.893 μV, and *angry* 4.620 μV) (see Figure 3A). The covariate Singing days reached close to a significant result, [*F*(1, 19) = 4.195, *p* = 0.055, η^2^ = 0.181].

In the second latency window 400–500 ms, the rmANOVA revealed a main effect of Group [*F*(1, 36) = 5.367, *p* = 0.026, η^2^ = 0.130], as the responses of the singing group infants were statistically larger and differed from those of the control group infants’ responses (singing intervention group 3.248 μV; control group 1.378 μV). There was also a main effect of Stimulus [*F*(8, 29) = 18.558, *p* ≤ 0.001, η^2^ = 0.340], as the emotional sounds statistically differed from the phonetic deviants (see Supplementary Table 6). Interaction effect was found for Sex and Group [*F*(1, 36) = 7.036, *p* = 0.012, η^2^ = 0.163], as the responses of the female infants were statistically larger than those of the male infants in the intervention group, *p* = 0.008 (singing intervention females 4.891 μV; singing intervention males 1.605 μV). Additionally, there was an interaction effect for Group and Stimulus [*F*(8, 29) = 2.958, *p* = 0.018, η^2^ = 0.076], resulting from the singing group eliciting larger response for the emotional stimuli *happy, p* = 0.028 (singing intervention group 7.104 μV; control group 2.962 μV), and *angry, p* = 0.010 (singing intervention group 7.838 μV; control group 3.318 μV) than the control group. The rmANOVA also showed an interaction effect for Sex and Stimulus [*F*(8, 29) = 3.158, *p* = 0.013, η^2^ = 0.081], as a result of the responses for emotional stimulus *angry* were larger in the female infants when compared to the male infants, *p* = 0.017 (females 7.663 μV; males 3.493 μV).

After controlling for the confounding variable Singing days in the rmANCOVA in the latency window 400–500 ms, the results revealed a main effect [*F*(1, 19) = 10.535, *p* = 0.004, η^2^ = 0.357], indicating that the duration of the singing intervention was positively associated with the response amplitudes. Thus, those preterm infants in the singing intervention group that were exposed to a longer singing intervention in days elicited larger MMRs (Figure 3B). Additionally, a main effect of Sex was found, [*F*(1, 19) = 5.680, *p* = 0.028, η^2^ = 0.230], resulting from the female preterm infants having the largest MMRs (adjusted means: singing intervention females 4.227 μV, singing intervention males 1.800 μV). The results also showed an interaction effect of Stimulus and Sex, [*F*(8, 12) = 3.610, *p* = 0.010, η^2^ = 0.160], due to female infants’ MMRs being larger for the emotional sound *angry, p* = 0.009 (adjusted means: singing intervention females 11.752 μV, singing intervention males 3.695 μV).

**FIGURE 4:**
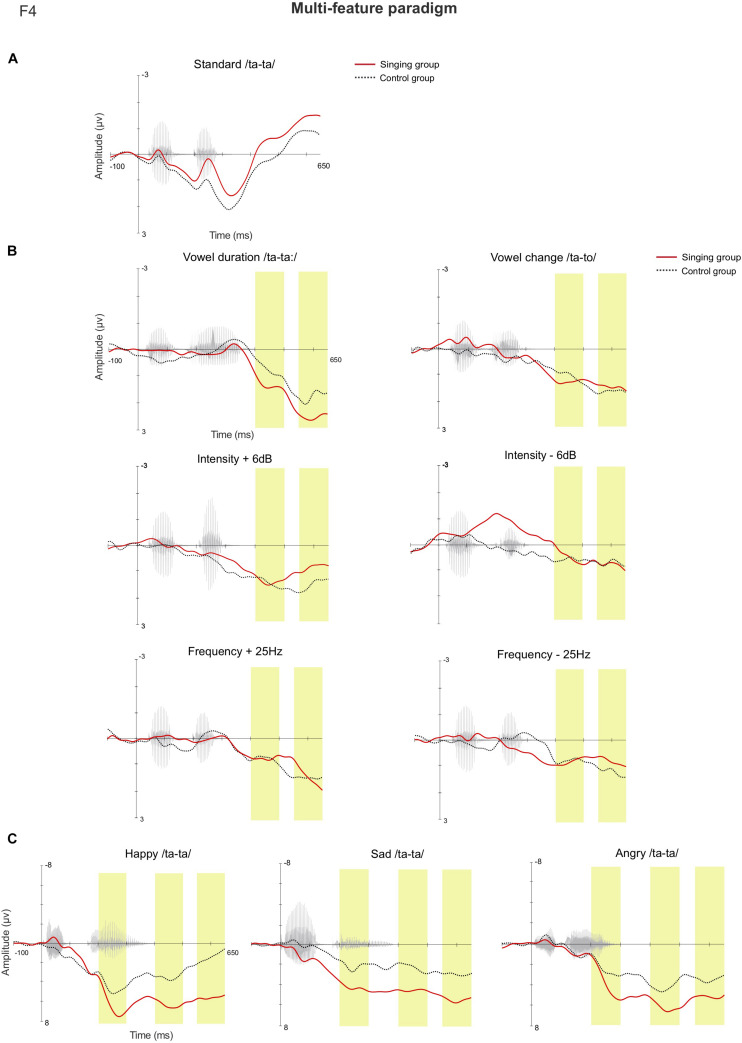
The AERP waveforms between the singing intervention and control group in the multi-feature paradigm at the electrode F4 for **(A)** the standard stimulus, **(B)** the standard-subtracted waveforms for both the phonetic deviants, and **(C)** the emotional stimuli. The yellow bars highlight the examined latency windows 200–300, 400–500, and 550–650 ms.

In the late latency window 550–650 ms, rmANOVA revealed a main effect of Group [*F*(1, 36) = 4.405, *p* = 0.043, η^2^ = 0.109], as the mean magnitude of the MMRs were larger in the singing intervention group when compared to the control group (singing intervention group, 3.018 μV, control group, 1.369 μV). The responses to emotional sounds were statistically larger than responses to the phonetic deviants, leading to a main effect of Stimulus [*F*(8, 29) = 13.849, *p* ≤ 0.001, η^2^ = 0.278] (see detailed *p*-values in Supplementary Table 6). An interaction effect between Sex and Group [*F*(1,36) = 5.169, *p* = 0.029, η^2^ = 0.126] was discovered, as the responses of female infants were statistically larger than those of the male infants in the singing intervention, *p* = 0.042 (singing intervention females, 4.219 μV; singing intervention males, 1.817 μV). An interaction effect between Stimulus and Group [*F*(8, 29) = 3.776, *p* = 0.004, η^2^ = 0.095] was found, due to responses to the emotional sounds being larger in the singing intervention group: *happy, p* = 0.010 (singing intervention group, 5.840 μV, control group, 1.178 μV); *sad, p* = 0.021 (singing intervention group, 5.871 μV, control group, 2.873 μV); and *angry*, *p* = 0.045 (singing intervention group, 6.022 μV, control group, 3.016 μV). Figure 4 illustrates the AERP waveforms in both groups for all the deviant stimuli at the electrode F4.

The rmANCOVA analysis revealed a main effect of the covariate Singing days [*F*(1, 19) = 7.541, *p* = 0.013, η^2^ = 0.284] in the latency window 550–650 ms, meaning that the singing intervention duration in days was positively associated with the magnitude of the MMRs (Figure 3C). A main effect of Stimulus was discovered, [*F*(8, 12) = 5.212, *p* = 0.001, η^2^ = 0.215], as a result of the emotional sounds differing from the phonetic deviants (see Supplementary Table 7). Finally, an interaction effect of Stimulus and Sex was revealed, [*F*(8, 12) = 2.666, *p* = 0.037, η^2^ = 0.123], when responses of the female preterm infants were larger than those of the male preterm infants for the phonetic deviant *vowel duration, p* = 0.045 (adjusted means: singing intervention females 3.931, singing intervention males 1.436), and emotional sound *sad, p* = 0.025 (adjusted means: singing intervention females 7.851 μV, singing intervention males 3.826 μV).

#### Oddball Paradigm

**FIGURE 5:**
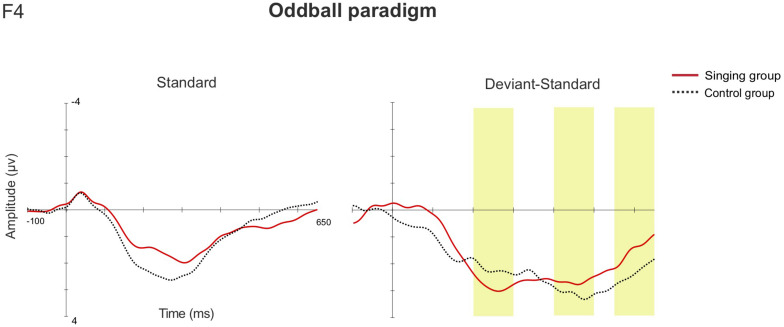
The AERP waveforms for the standard stimulus, and the standard-subtracted waveforms for the deviant stimulus between the singing intervention and control group in the oddball paradigm (F4). The yellow bars highlight the examined latency windows 200–300, 400–500, and 550–650 ms.

In the one-deviant oddball paradigm, MMRs were elicited in both groups in all three studied latency windows (The results reported in detail in Supplementary Table 3). In the early (200–300 ms) and later (550–650 ms) latency windows, rmANOVA showed no statistically significant main effects nor interaction effects between the different factors. In the second latency window, 400–500 ms, the main effect of Sex [*F*(1, 36) = 4.876, *p* = 0.034, η^2^ = 0.119] was discovered, as the responses of the female infants were statistically larger than those of the male infants (females 4.126 μV, males 1.621 μV). The rmANCOVA analysis showed no relation between the magnitude of the MMRs and the overall singing duration in any of the latency windows. The AERP waveforms from both groups at the electrode F4 are shown in Figure 5.

## Discussion

We examined whether parental singing during kangaroo care enhances the neural processing of phonetic and emotional speech sounds in preterm infants at term age. The results showed group differences in the discrimination of the speech sound changes in all three studied latency windows with larger MMRs elicited in infants exposed to parental singing than in control infants. The group differences were due to larger MMRs evoked to the emotional speech sounds in the singing intervention group and the largest MMRs elicited by the preterm female infants. Nonetheless, the overall singing intervention duration in days was positively associated with the magnitude of the MMRs in both sexes in the majority of the studied latency windows. Thus, the more singing days were conducted, the larger MMRs were elicited for both phonetic and emotional speech sounds in both preterm male and female infants. Daily singing time (min) was not connected with the MMR amplitudes, indicating that especially repeated daily exposure to singing affected the MMRs more than prolonged short-term singing. In the oddball paradigm, MMRs to the deviant change were elicited in both groups, and no group differences for the non-speech sounds were found. Also, no relation between the daily singing time nor the overall singing intervention duration and the response amplitudes for the non-speech sounds were discovered.

These results demonstrate that parental singing during kangaroo care may improve the discrimination of phonetic and emotional speech sounds in preterm infants. In particular, frequent exposure to parental singing may enhance the change-detection processing of speech sounds, whereas short-term singing even for extended periods may have only a small effect or no effect at all. The results from the oddball paradigm showing no group differences or relations between the singing time and the response amplitudes for the non-speech sounds strengthens the conclusion that parental singing during kangaroo care improved, especially the neural processing of speech sound changes in preterm infants.

During the early postpartum weeks after preterm birth, infants are mainly exposed to the sounds in the NICU, such as the monitor alarm sounds and silence rather than adult language (Caskey et al., 2011). At an early developmental period, the rat cortical sensory neurons have been shown to respond to the stimuli that occur more frequently in the surrounding environment (Han et al., 2007). Thus, the development of the auditory system might be promoted when the hospital’s sound environment would consist of developmentally supportive auditory stimuli, such as parental voice, speech, and singing to help preterm infants’ auditory cortices tune more on the developmentally appropriate stimuli. Carvalho et al. (2019) investigated the effects of maternal ID singing and speech during kangaroo care on vocalization in preterm infants in the NICU. Their results showed a decrease in infant vocalization during both conditions interpreted as an increased level of attention. In turn, in a study by Caskey et al. (2014), exposure to parent talk during neonatal intensive care was associated with higher Bayley language and cognitive scores at 7- and 18-month corrected age in preterm infants. Nonetheless, Coppola and Cassibba (2010) showed that mothers talk less on the ward if their preterm infant’s medical state is severe. For these reasons, parents should be informed about the importance of using their voices (speaking, singing, and humming) when their preterm infant is cared for in neonatal units.

In our previous study, we reported that the mothers who participated in the singing intervention group of this study benefitted from being guided by a specially trained music therapist about how to use their voices age-appropriately in a live interaction with their preterm infant during their hospital stay (Kostilainen et al., 2021). The results also showed that singing during kangaroo care was mostly experienced as a natural way to be in contact, and it enhanced wellbeing by decreasing maternal anxiety and by improving mood. Singing during kangaroo care also supported the mother-infant relationship by creating interactive moments and promoting emotional connection (Kostilainen et al., 2021). Singing during kangaroo care may thus offer pervasive support after preterm birth by improving not only infant auditory processing but also maternal wellbeing, and the development of their early relationship.

The emotional stimuli in the multi-feature paradigm elicited the most prominent MMRs in both groups. The happy-sounding stimulus elicited the largest MMRs, as also found in the previous study on full-term infants (Kostilainen et al., 2018). The results of the current study revealed a group difference for the emotional sounds in the latency windows 400–500 and 550–650 ms, as the singing intervention group infants had larger MMRs to the emotional sounds when compared to the control group infants. Also, the overall singing intervention duration was positively associated with the magnitude of the MMRs for the emotional sounds *happy* and *sad* in the first latency window 200–300 ms, yet not for the emotional sound *angry* in any of the latency windows. As our previous results from the Singing Kangaroo study showed that mothers experienced singing during kangaroo care to enhance emotional closeness (Kostilainen et al., 2021), it would be interesting to study further the impact of parental singing on the neural processing of emotional sounds in particular.

Our data revealed several sex differences. Preterm female infants elicited larger MMRs than the male infants in the singing intervention group. After controlling for the singing intervention duration, the female infants elicited larger MMRs, confirming that the sex effect was not a result of female preterm infants being exposed to a more prolonged and intense singing intervention. These results suggest that parental singing during kangaroo care affected the auditory processing of speech sounds more efficiently in preterm females than in male infants. Furthermore, female infants in both groups elicited larger MMRs for the non-speech deviant change in the oddball paradigm when compared to the preterm male infants, indicating that auditory processing of simple tone sounds in female infants was more enhanced in general.

Sex differences have been reported in previous infant studies showing more enhanced auditory processing in females. Using transient otoacoustic emissions hearing screening, male full-term infants were discovered to have decreased levels of sensitivity to high frequencies when compared to those of female full-term infants (Cassidy and Ditty, 2001). Studies have also suggested that auditory perception of speech sounds in female infants (Mueller et al., 2012) and neurological and developmental integration of music stimuli are more enhanced in female preterm infants than in males (Walworth et al., 2012). In general, male preterm infants tend to have more adverse short- and long-term outcomes than preterm female infants that have been connected to hormonal, immunological, and genetic differences (O’Driscoll et al., 2018). Even though the underlying reasons for sex differences are not completely clear, differences in auditory processing seem to be a real effect, implying that our results regarding more enhanced processing of sounds in female preterm infants were not necessarily connected to the small sample size.

## Limitations

There were some limitations in this study. The intervention period was several weeks long, and it was independently carried out by the parents. After being discharged from the hospital, several parents reported challenges in conducting the intervention at home, especially those with twins or older siblings. Hence, many of the families were not able to continue the intervention to term age, and the number of infants measured with EEG at 40 GW was smaller than initially planned. It should be considered that the high drop-out rate that resulted in a small sample size reduced the statistical power and may have affected the results. Additionally, our results of the sex differences should be replicated in a larger sample, since besides the number of participants in our study was small, the sex distribution in the singing intervention group and between the groups was not well balanced. In further studies, attempts should also be made to adjust the intervention for higher compliance after discharge and in varying family situations.

Regarding the sound paradigms used in our study, infant studies assessing change detection assess MMRs by subtracting the standard response from that of the deviant response. However, response amplitudes between the standard and the deviant differ due to different presentation rates and in adults, this results in differences in, e.g., N1 response amplitudes. As a result, the standard-subtracted MMR waveform may contain activity not related to change detection. While adequate change-detection responses can be obtained even when presentation rates between the standard and the deviant differ (Jacobsen et al., 2003), the response amplitudes are confounded more the larger the difference between the standard and the deviant is (Horváth et al., 2008; Peter et al., 2010). However, it is not known how this effect is seen in infants. It could be argued that the effect could be largest for the emotional sounds as the acoustical difference from the standard is the largest, but deviance magnitude may not always influence the infant MMN (He et al., 2009). To summarize, there is a need for further studies on what types of confounding factors influence the infant MMRs and what the functional significance of the infant MMR is (Kushnerenko et al., 2013). It is likely that the effect of the singing intervention is mainly due to changes in the infant MMR, but without a control condition, we cannot rule out whether some of the effects could be due to responses in sound *per se*.

## Conclusion

In conclusion, considering that early auditory sensory experience affects preterm infant development, finding ways to promote neurodevelopment during the especially vulnerable period of brain development should be of high priority. Our results indicate that parental singing during kangaroo care may enhance the neural processing of emotional and phonetic speech sound changes in preterm infants at term age. Especially, frequent exposure to singing may improve the development of the auditory system more efficiently than short-term exposure. Thus, simple daily singing routines, such as singing during kangaroo care, singing lullabies when putting infants to sleep, or singing songs during a diaper change, feeding or bath, can be recommended for parents of preterm infants to support the development of the auditory system and improve the neural processing of speech sounds.

As accurate sound discrimination and the ability to process minor changes in speech is a requirement for normal language development, parental singing during kangaroo care could be used to promote speech sound discrimination abilities in preterm infants that are at risk for atypical neurodevelopment. Parental singing could offer a non-medical, non-invasive, and cost-effective way to support the development of the auditory system after preterm birth. Future studies investigating the long-term neurodevelopmental effects of parental singing, directed and non-directed musical activities, as well as music therapy on the development in preterm infants, would be needed.

## Data Availability Statement

The raw data supporting the conclusions of this article will be made available by the authors, without undue reservation.

## Ethics Statement

The studies involving human participants were reviewed and approved by Ethics Committee of Hospital District of Helsinki. Written informed consent to participate in this study was provided by the participants’ legal guardian/next of kin.

## Author Contributions

VF, MH, SP, KM, and KK designed and planned the study. KK recruited the families and guided parents to conduct the intervention, stored the data, analyzed the data, interpreted the data, and wrote the manuscript. MH supervised the work of KK. KM was the corresponding physician of the study in Jorvi hospital and arranged the settings for the EEG measurements. VW wrote the MATLAB script and co-operated the EEG processing together with KK. EP wrote the MATLAB script for the point-by-point *t*-tests and assisted KK in the analyses. All co-authors EP, KM, VW, SP, VF, and MH reviewed the manuscript. All authors contributed to the article and approved the submitted version.

## Conflict of Interest

The authors declare that the research was conducted in the absence of any commercial or financial relationships that could be construed as a potential conflict of interest.

## Publisher’s Note

All claims expressed in this article are solely those of the authors and do not necessarily represent those of their affiliated organizations, or those of the publisher, the editors and the reviewers. Any product that may be evaluated in this article, or claim that may be made by its manufacturer, is not guaranteed or endorsed by the publisher.
