# Supplementary material for: Repeated Parental Singing During Kangaroo Care Improved Neural Processing of Speech Sound Changes in Preterm Infants at Term Age
Source: Front Neurosci. 2021 Sep 3;15:686027. doi: 10.3389/fnins.2021.686027 (PMC8446605; doi:10.3389/fnins.2021.686027)
Supplement: Supplementary file 1 [file Data_Sheet_1.docx]

Supplementary Material

**Supplementary Figure 1.** Spectrograms of the standard, deviant and emotional stimuli in the multi-feature paradigm (same as in Kostilainen et al., 2020).

a) Standard (/ta-ta/)


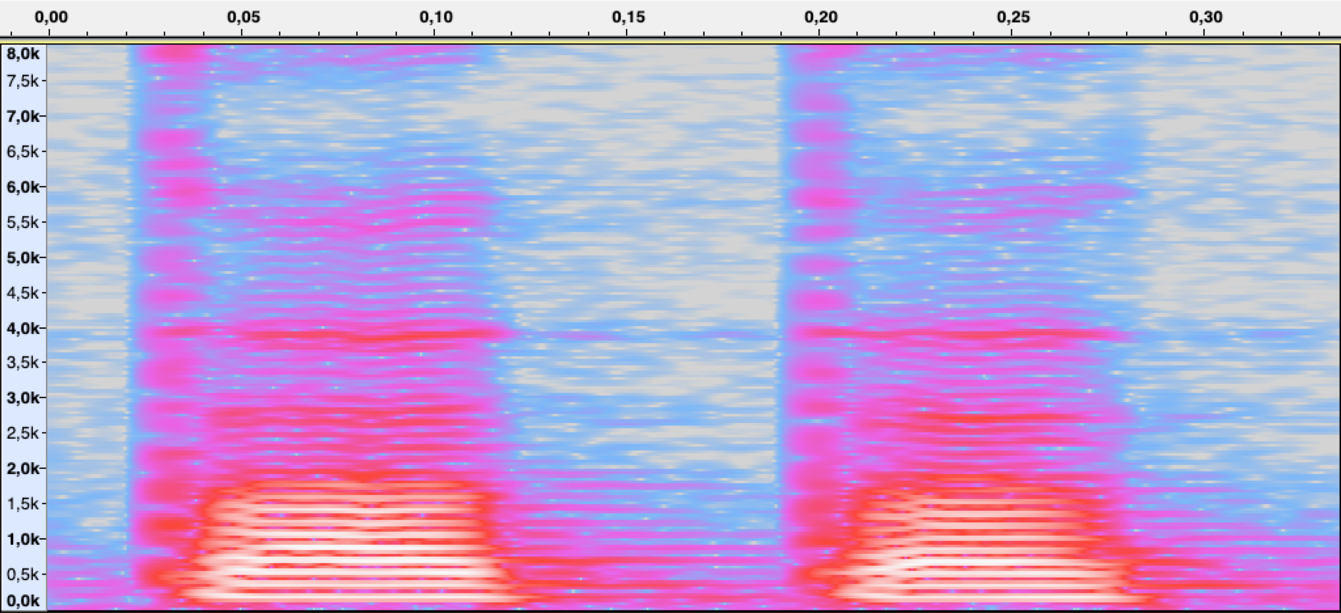


b) Vowel duration (/ta-ta:/)


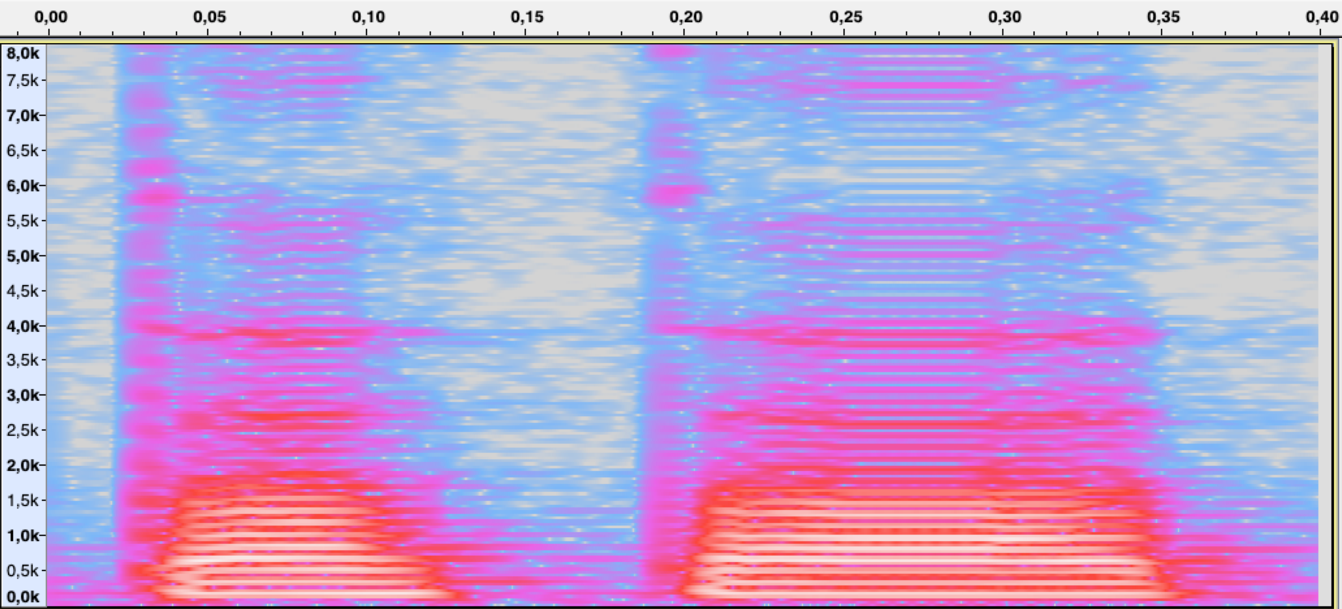


c) Vowel change (/ta-to/)


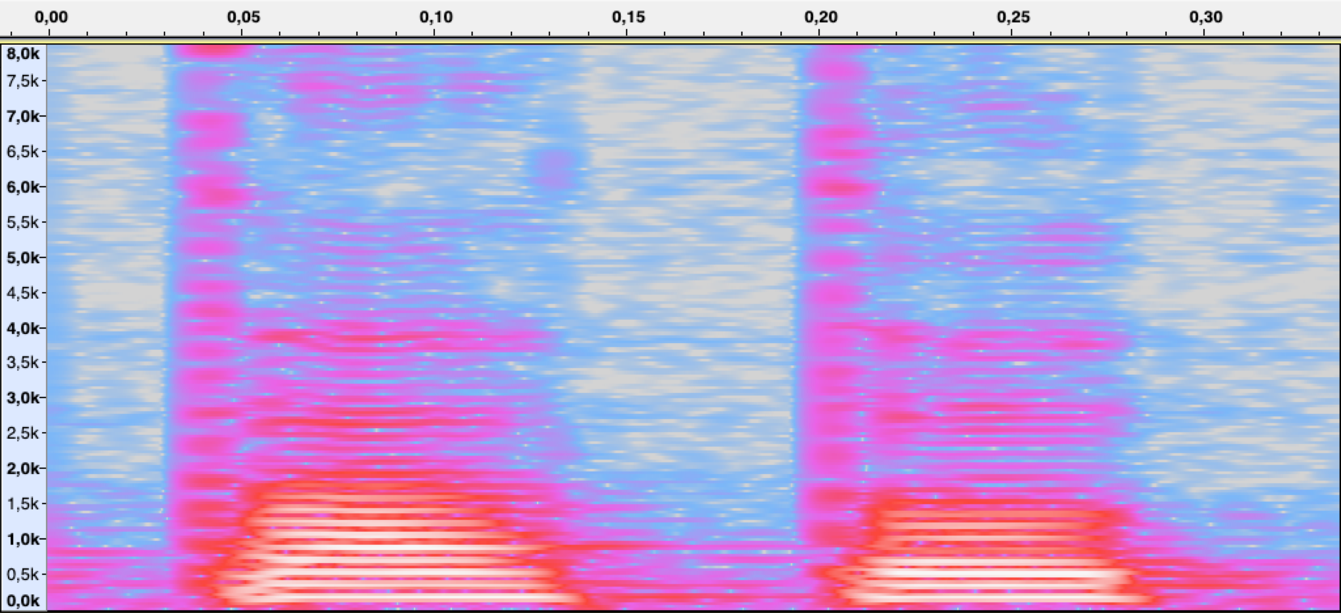


d) Intensity change (+6 dB)


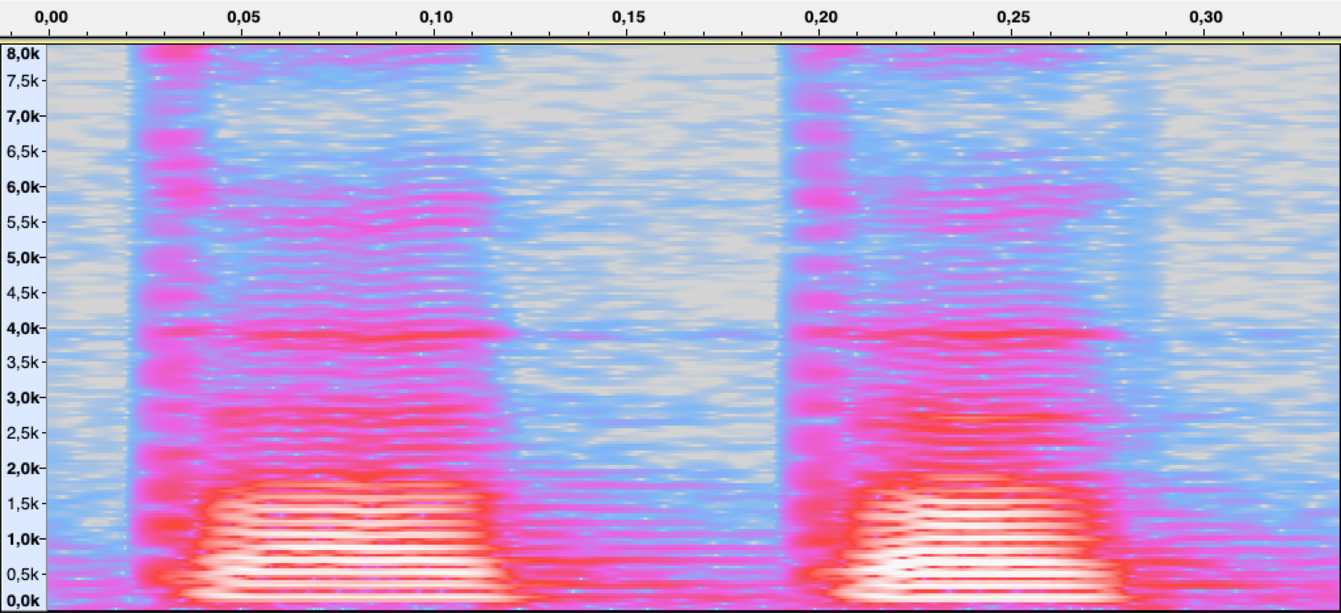


e) Intensity change (-6 dB)


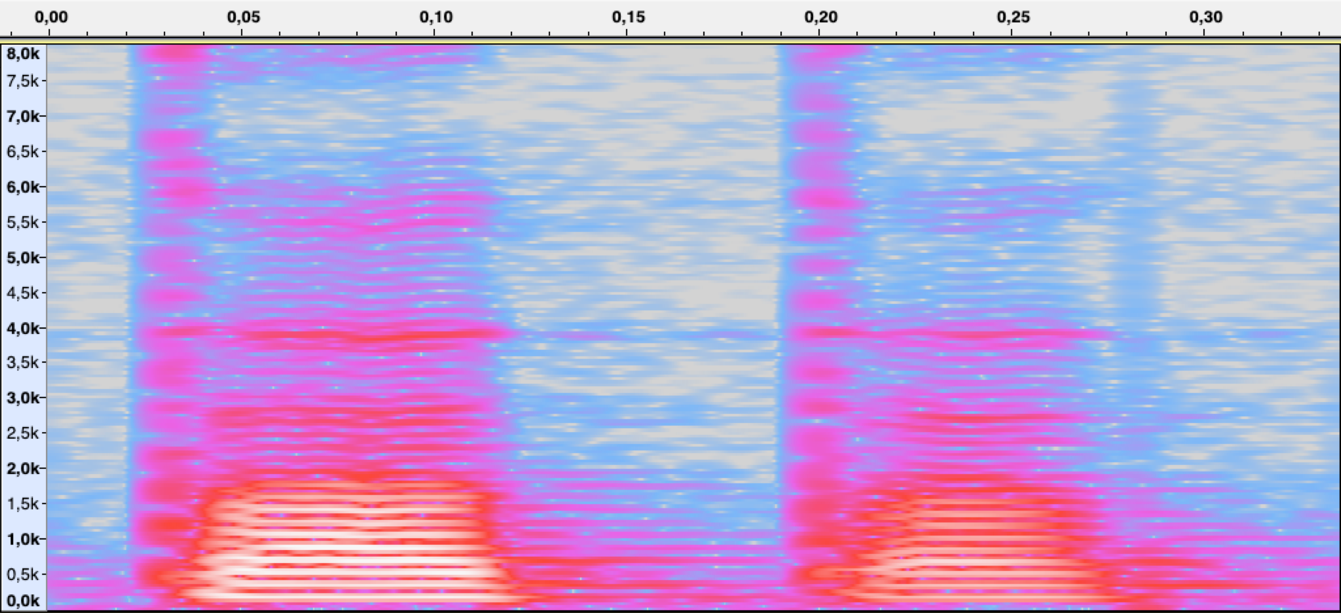


f) Frequency change (+25.5 Hz)


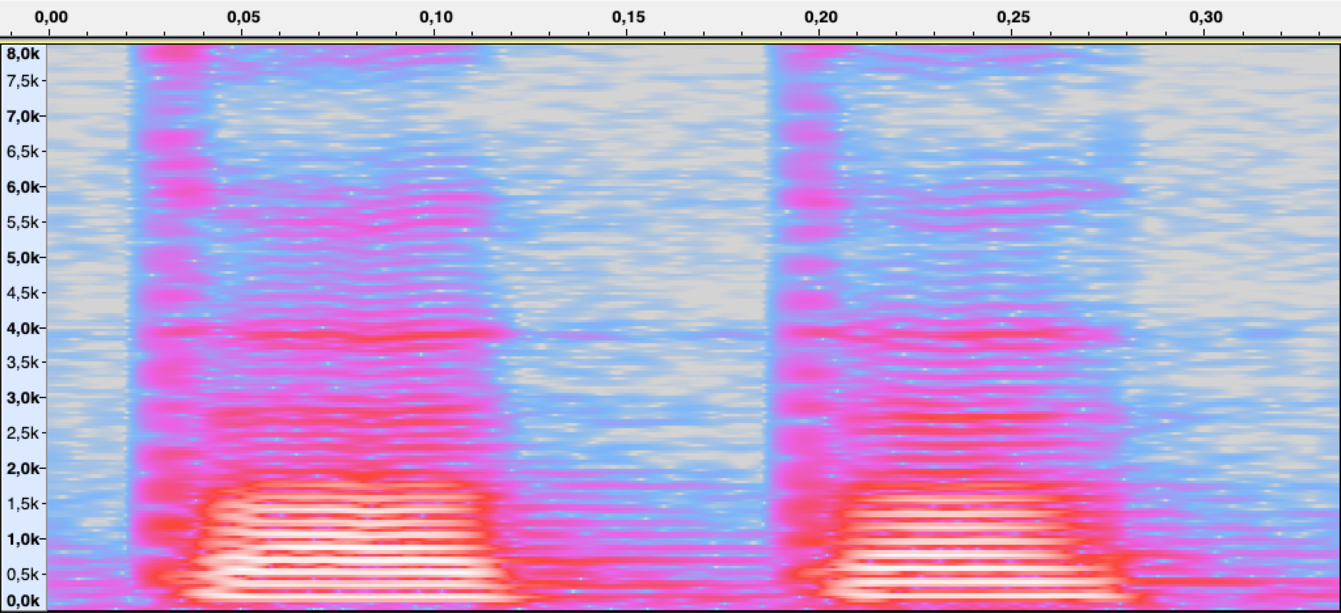


g) Frequency change (-25.5 Hz)


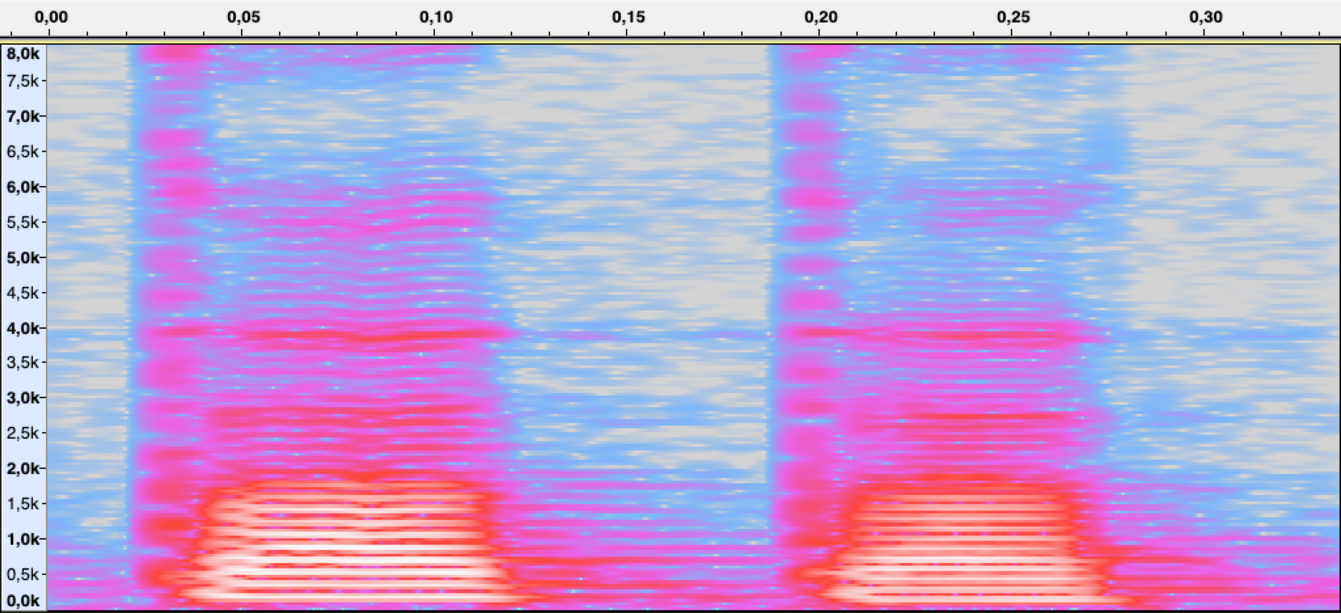


h) Happy (/ta-ta/)


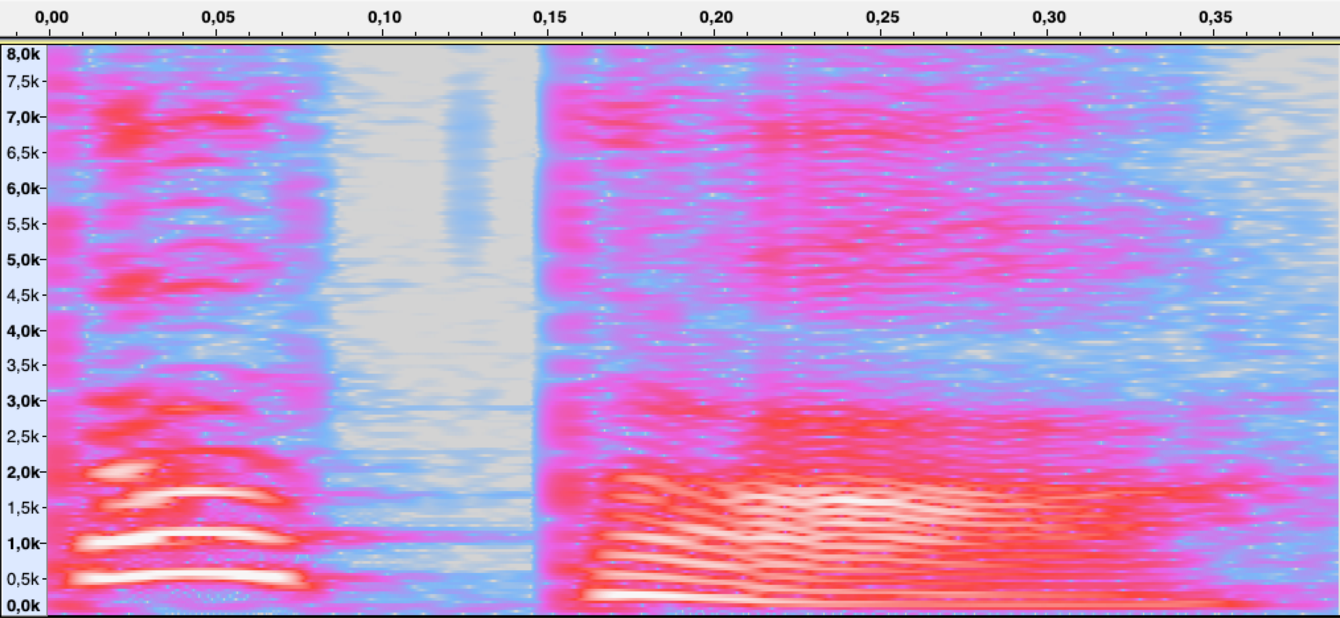


i) Sad (/ta-ta/)


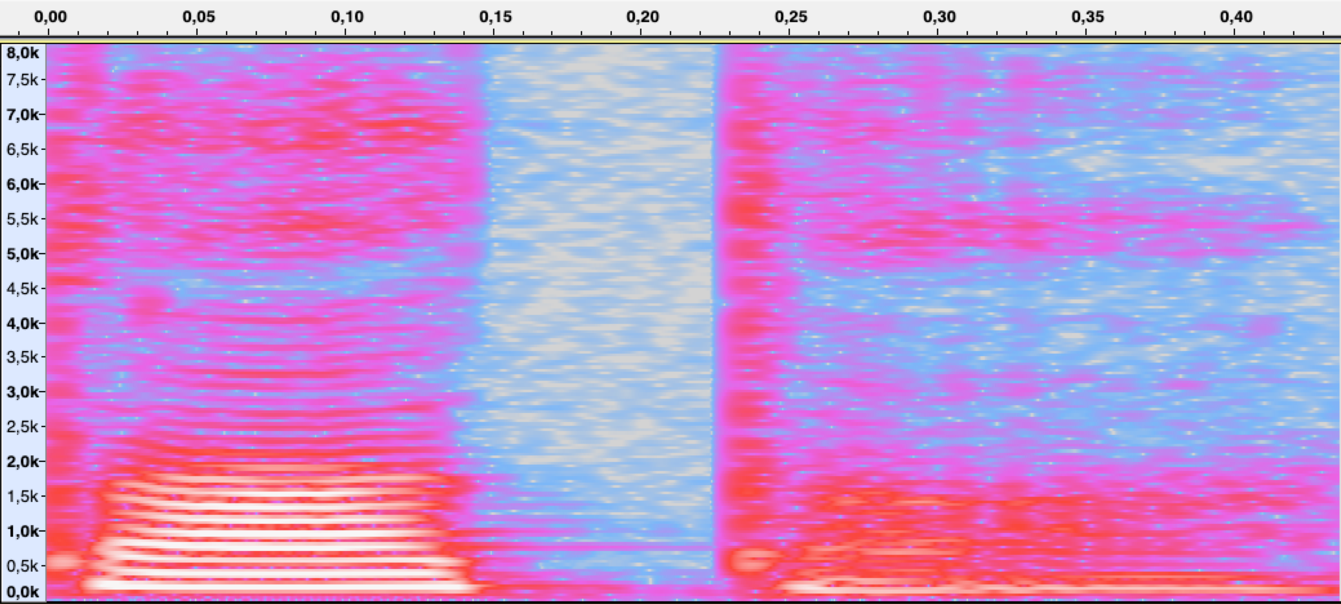


j) Angry (/ta-ta/)


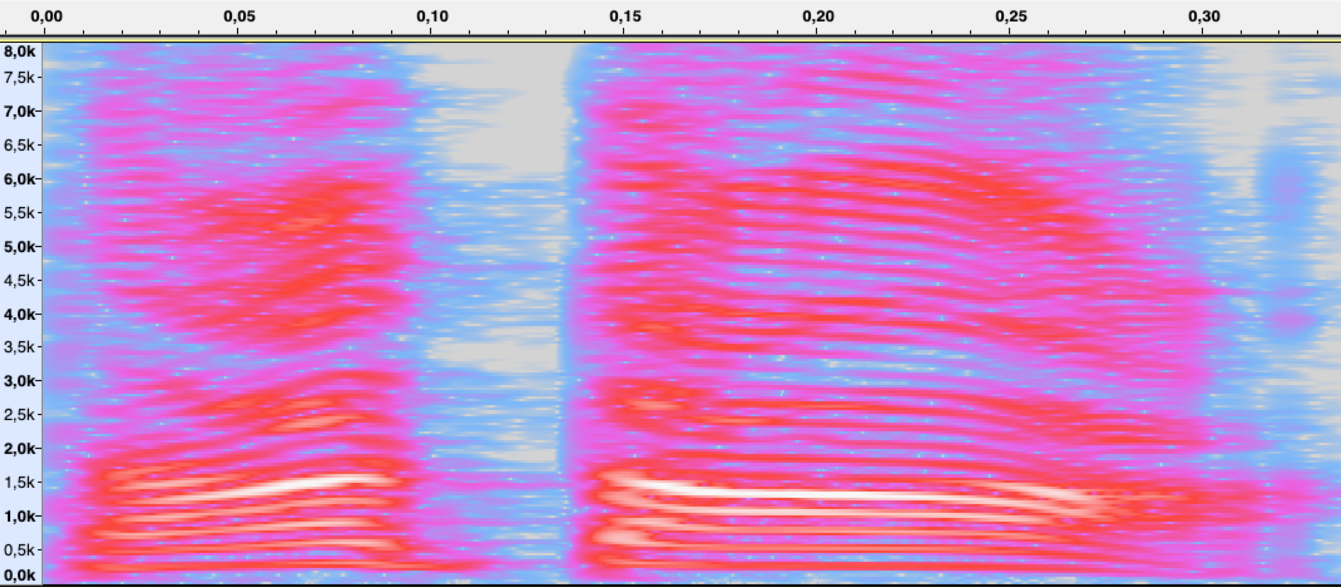


**Supplementary Table 1.** The recognition test results of the emotional sounds used in the multi-feature paradigm. Adult participants (n = 5) listened to the recording and chose the closest emotion from the five basic emotion list (happiness, anger, fear, sadness, shame) that in their opinion corresponded best to the emotional sound in question. If the participants were unable to recognize the emotion, they were instructed to choose the two most suitable ones (Same as in Kostilainen et al., 2020).

| Target sound | *Happy* | *Sad* | *Angry* |
| --- | --- | --- | --- |
| Recognized emotions |  |  |  |
| Happiness | 100% | 0% | 0% |
| Anger | 0% | 0% | 100% |
| Fear | 0% | 0% | 0% |
| Sadness | 0% | 75% | 0% |
| Shame | 0% | 17% | 0% |
| Both sadness and shame | 0% | 8% | 0% |

**Supplementary Figure 2.** Multi-feature paradigm.

a) *t*-tests for each timepoint of the whole auditory ERP signal in the singing intervention preterm group (electrodes F3, F4, C3 and C4 combined). The dotted line representes the limit of significance, *p* = < 0.05.


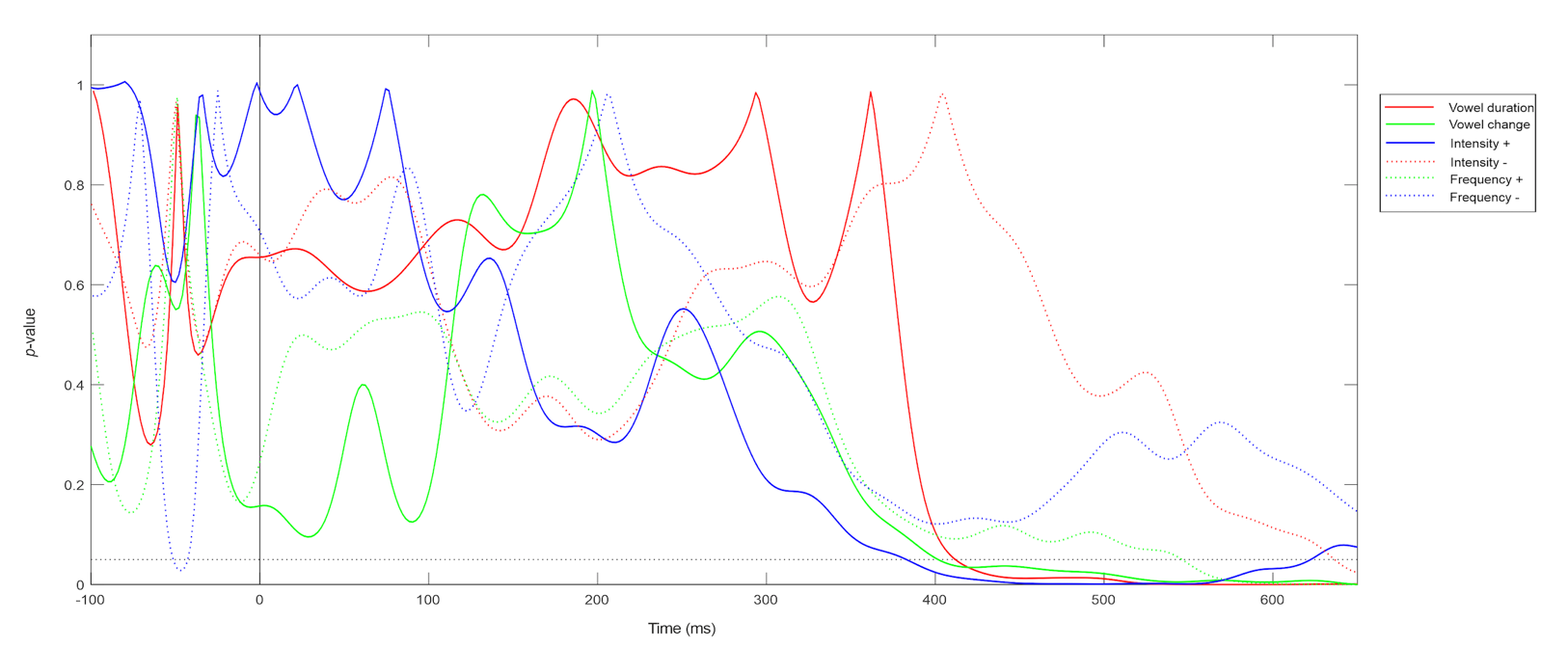

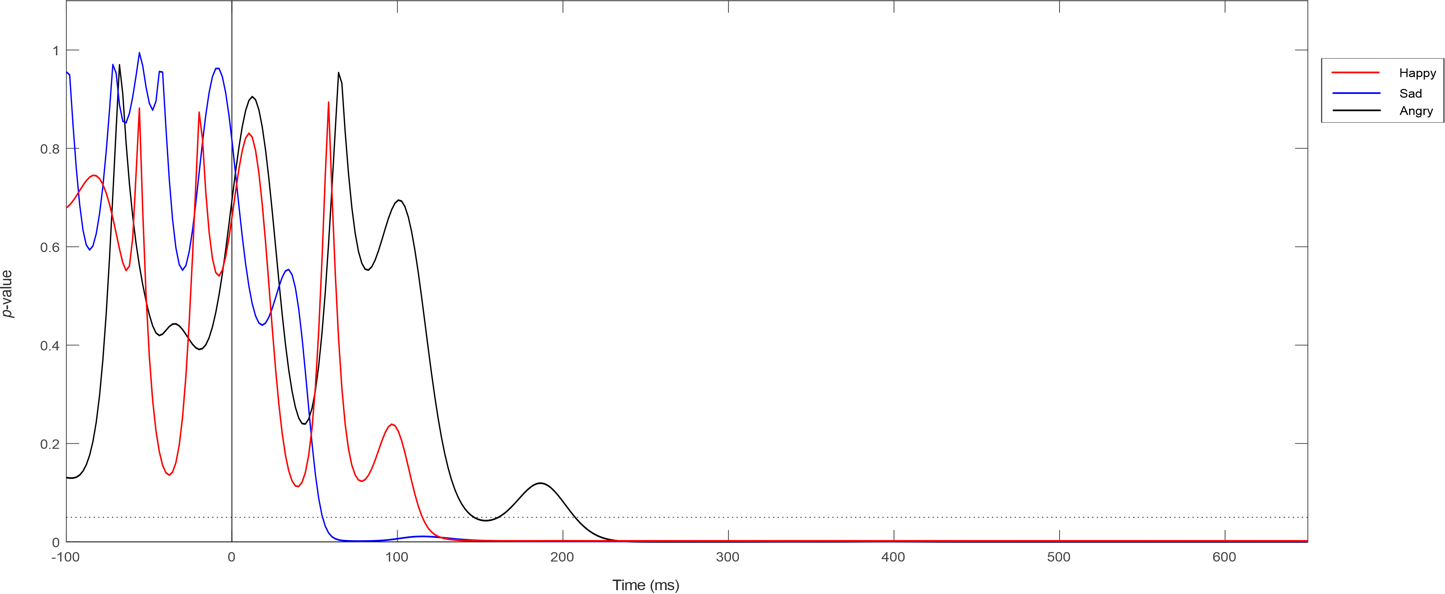


b) *t*-tests for each timepoint of the whole auditory ERP signal in the control preterm group (electrodes F3, F4, C3 and C4 combined). The dotted line representes the limit of significance, *p* = < 0.05.

**
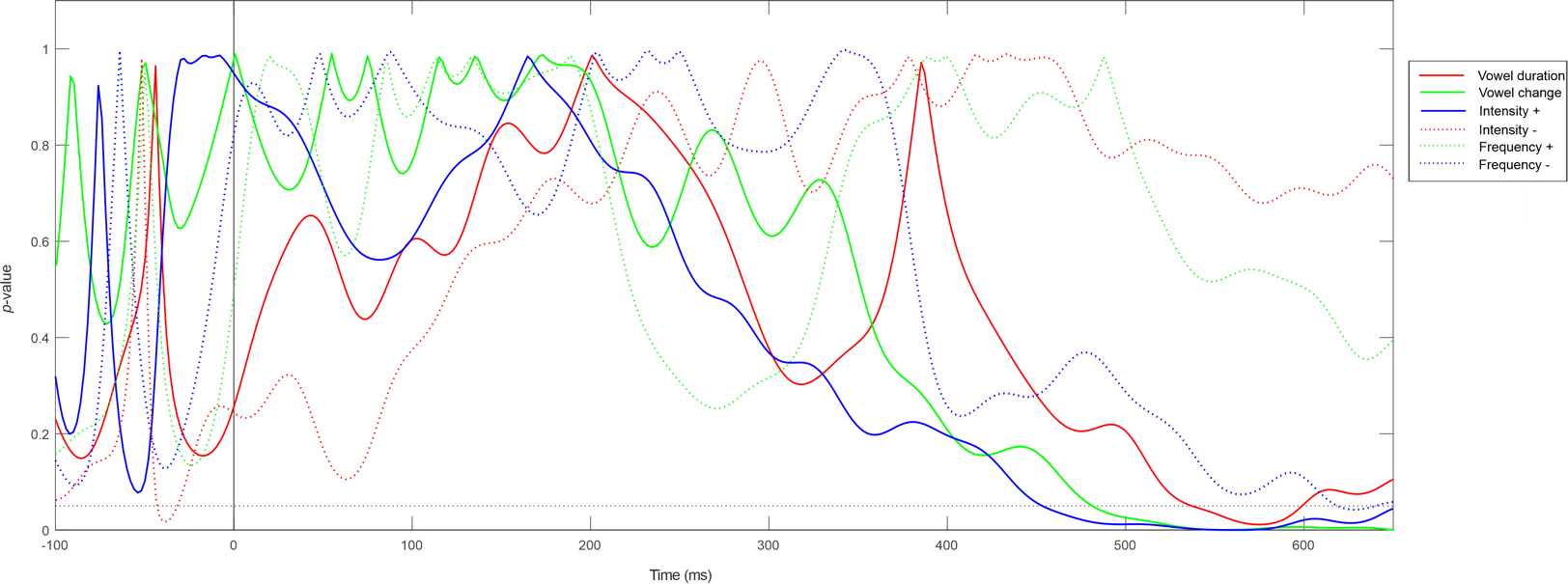
**


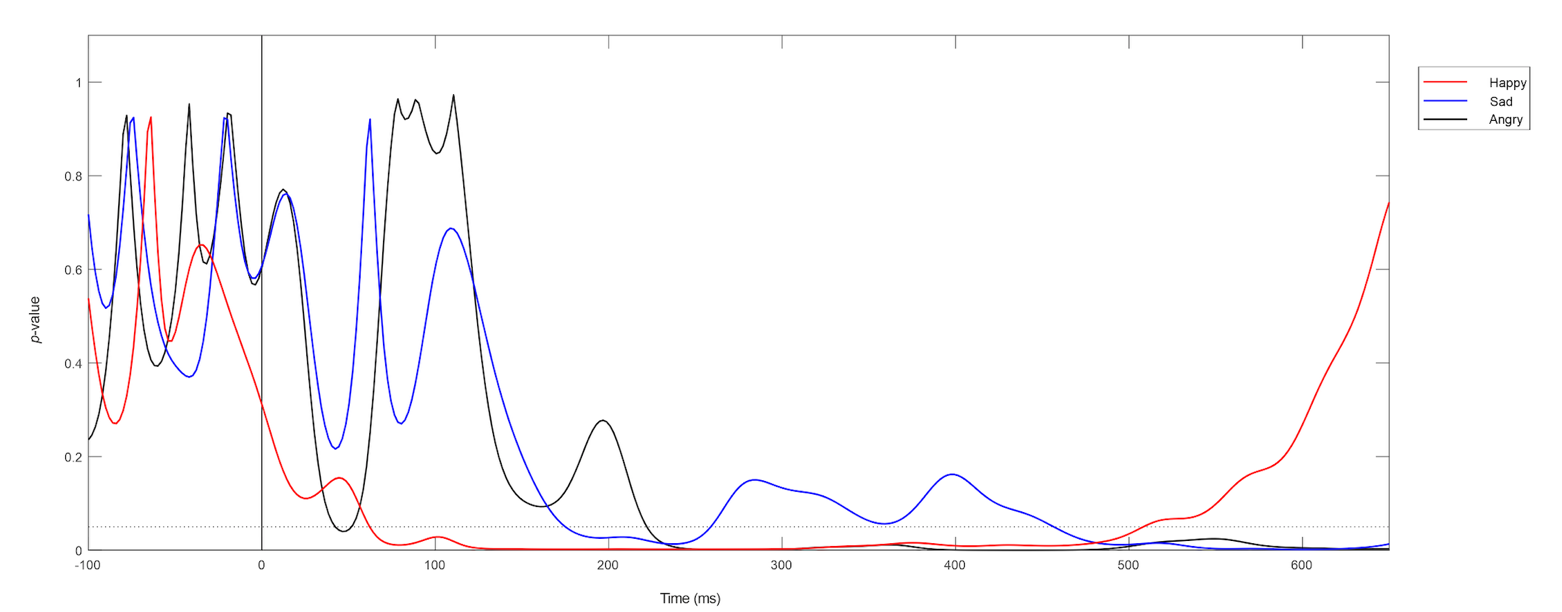


**Supplementary Figure 3.** Oddball paradigm.

a) *t*-tests for each timepoint of the whole auditory ERP signal in the singing intervention group (electrodes F3, F4, C3 and C4 combined). The dotted line representes the limit of significance, *p* = < 0.05.


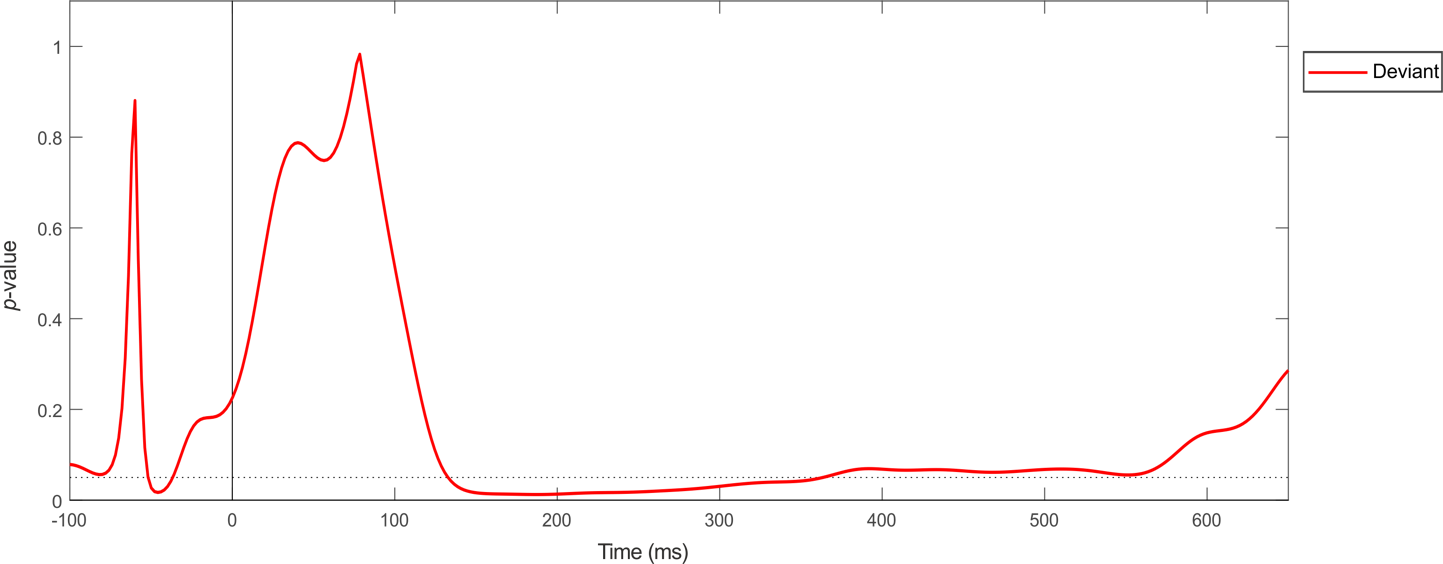


b) *t*-tests for each timepoint of the whole auditory ERP signal in the control group (electrodes F3, F4, C3 and C4 combined). The dotted line representes the limit of significance, *p* = < 0.05.


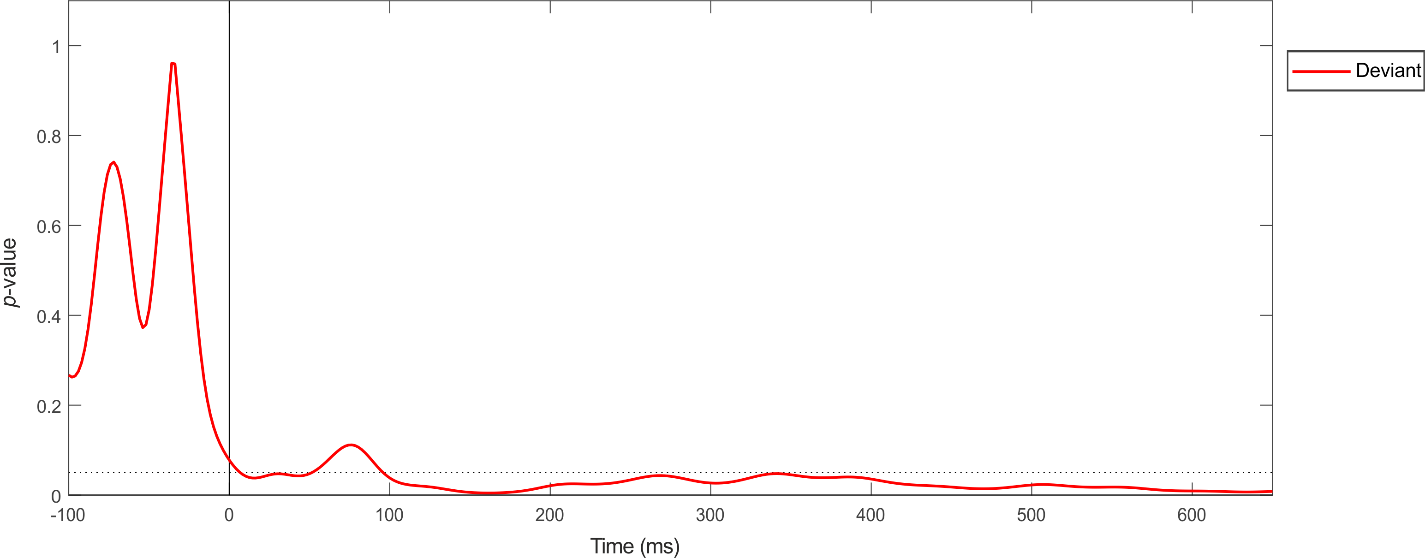


**Supplementary Table 2.** The birth characteristics of the preterm infants (mean and range) according to the recruiting hospital. No statistically significant differences between the two neonatal wards were discovered.

|  | Jorvi Hospital | Kätilöopisto Maternity Hospital |
| --- | --- | --- |
| Preterm infants*, n* | 23 | 17 |
| Male (%) | 14 (61%) | 10 (59%) |
| Gestational weeks at birth | 30.2 (26.7–33.3) | 30.9 (27.1–33.3) |
| Weight (g) | 1444.1 (900–2800) | 1448 (925–1950) |
| Height (cm) | 40 (35–48) | 40 (36–45) |
| SGA^a^ | 2 | 4 |
| Umbilical cord arterial pH | 7.25 (6.98–7.38) | 7.25 (7.11–7.34) |
| Apgar 5 min^b^ | 6.6 (2–10) | 7 (1–9) |

^a^ Born as small for gestational age, birth weight of less than -2 standard deviations for the age. ^b^Newborn health assessment on a scale 1–10, assessed 5 min postnatally (Breathing effort, heart rate, muscle tone, reflexes and skin color)

**Supplementary Table 3.** Results of the one-sample *t*-tests (*t*- and *p*-values, means, standard deviations, and 95 % confidence intervals).

a) Multi-feature paradigm

|  |  |  | *t* sig | MEAN (SD)  *(CI)* |  |  |  |  |
| --- | --- | --- | --- | --- | --- | --- | --- | --- |
| Electrode | F3 |  | F4 |  | C3 |  | C4 |  |
| Singing intervention group  LATENCY WINDOW  200–300 MS |  |  |  |  |  |  |  |  |
| Emotional stimulus |  |  |  |  |  |  |  |  |
| *Happy* | 5.5*** | 6.1 (5.1)  *(+3.78… 8.34)* | 5.6*** | 6.2 (5.2)  *(+3.87… 8.51)* | 4.6*** | 5.5 (5.5)  *(+3.00… 7.90)* | 3.8*** | 4.4 (5.4)  *(+1.96… 6.79)* |
| *Sad* | 4.2*** | 4.1 (4.6)  *(+2.08… 6.19)* | 4.2*** | 4.4 (4.9)  *(+2.25… 6.58)* | 3.6** | 3.8 (5.0)  *(+1.63… 6.04)* | 3.4** | 3.1 (4.3)  *(+1.23… 5.03)* |
| *Angry* | 2.9** | 3.3 (5.3)  *(+0.94… 5.63)* | 3.5** | 3.5 (4.7)  *(+1.43… 5.63)* | 3.5** | 3.4 (4.5)  *(+1.36… 5.36)* | 4.7*** | 2.9 (2.9)  *(+1.62… 4.18)* |
| LATENCY WINDOW  400–500 MS |  |  |  |  |  |  |  |  |
| Emotional stimulus |  |  |  |  |  |  |  |  |
| *Happy* | 5.1*** | 6.3 (5.8)  *(+3.74… 8.88)* | 5.6*** | 6.3 (5.2)  *(+3.98… 8.63)* | 4.4*** | 5.6 (5.9)  *(+2.93… 8.17)* | 3.3** | 4.1 (5.8)  *(+1.50… 6.63)* |
| *Sad* | 4.0*** | 4.4 (5.1)  *(+2.14… 6.69)* | 4.1*** | 4.7 (5.3)  *(+2.32… 7.04)* | 3.9*** | 3.9 (4.6)  *(+1.83… 5.92)* | 3.4** | 3.5 (4.8)  *(+1.34… 5.61)* |
| *Angry* | 4.2*** | 6.1 (6.8)  *(+3.12… 9.15)* | 4.2*** | 5.9 (6.7)  *(+2.95… 8.89)* | 3.8*** | 5.7 (7.0)  *(+2.57… 8.81)* | 3.7*** | 4.3 (5.4)  *(+1.93… 6.76)* |
| Deviant stimulus |  |  |  |  |  |  |  |  |
| *Vowel duration* | 1.5 | 0.8 (2.3)  *(-0.28… +1.77)* | 2.2* | 1.3 (2.8)  *(+0.07… 2.55)* | 3.0** | 1.4 (2.2)  *(+0.43… 2.35)* | 2.6* | 1.5 (2.7)  *(+0.28… 2.64)* |
| *Vowel change* | 1.0 | 0.6 (2.8)  *(-0.65… +1.82)* | 2.1* | 1.3 (2.9)  *(+0.01… 2.56)* | 2.0 | 1.2 (2.8)  *(-0.06… +2.43)* | 2.5* | 1.6 (3.0)  *(+0.28… 2.96)* |
| *Intensity change*  *(+6 dB)* | 3.0** | 1.4 (2.2)  *(+0.44… 2.43)* | 2.4* | 1.6 (2.2)  *(+0.23… 2.93)* | 3.7*** | 1.8 (2.4)  *(+0.79… 2.89)* | 2.2* | 1.3 (2.7)  *(+0.07… 2.50)* |
| *Intensity change*  *(-6 dB)* | 0.5 | 0.3 (2.4)  *(-0.79… +1.36)* | 0.8 | 0.4 (2.3)  *(-0.63… +1.40)* | 0.9 | 0.6 (3.2)  *(-0.82… +1.99)* | -0.1 | -0.1 (2.4)  *(-1.13… +0.99)* |
| *Frequency change*  *(+25.5 Hz)* | 1.0 | 0.6 (2.9)  *(-0.67… +1.86)* | 1.1 | 0.7 (2.9)  *(-0.59… +1.94* | 2.0 | 1.1 (2.5)  *(-0.02… +2.15)* | 1.7 | 1.1 (3.1)  *(-0.22… +2.48)* |
| *Frequency change*  *(-25.5 Hz)* | 1.4 | 0.9 (2.8)  *(-0.38… +2.10)* | 1.4 | 0.9 (3.0)  *(-0.46… +2.22)* | 0.9 | 0.7 (3.5)  *(-0.87… +2.22)* | 1.5 | 1.0 (3.1)  *(-0.35… +2.39)* |
| LATENCY WINDOW  550–650 MS |  |  |  |  |  |  |  |  |
| Emotional stimulus |  |  |  |  |  |  |  |  |
| *Happy* | 4.6*** | 5.3 (5.5)  *(+2.91… 7.78)* | 5.4*** | 5.6 (4.8)  *(+3.42… 7.67)* | 3.9*** | 4.6 (5.6)  *(+2.13… 7.06)* | 3.1** | 3.6 (5.5)  *(+1.20… 6.04)* |
| *Sad* | 6.0*** | 5.0 (3.9)  *(+3.27… 6.72)* | 6.7*** | 5.7 (4.0)  *(+3.89… 7.44)* | 5.6*** | 4.5 (3.8)  *(+2.83… 6.19)* | 5.1*** | 3.8 (3.4)  *(+2.25… 5.30)* |
| *Angry* | 5.7*** | 5.5 (4.5)  *(+3.48… 7.46)* | 5.3*** | 5.0 (4.4)  *(+2.99… 6.89)* | 4.6*** | 5.2 (5.3)  *(+2.84… 7.51)* | 4.1*** | 3.7 (4.2)  *(+1.83… 5.56)* |
| Deviant stimulus |  |  |  |  |  |  |  |  |
| *Vowel duration* | 2.7* | 1.9 (3.3)  *(+0.40… 3.35)* | 3.3** | 2.5 (3.5)  *(+0.90… 4.02)* | 2.9** | 1.9 (3.1)  *(+0.52… 3.28)* | 2.6* | 1.8 (3.1)  *(+0.36… 3.15)* |
| *Vowel change* | 1.5 | 0.9 (2.9)  *(-0.35… +2.23)* | 2.5* | 1.5 (2.8)  *(+0.27… 2.72)* | 1.6 | 1.0 (2.9)  *(-0.31… +2.21)* | 2.1* | 1.3 (2.8)  *(+0.01… 2.51)* |
| *Intensity change*  *(+6 dB)* | 1.7 | 1.0 (2.7)  *(-0.22… +2.17)* | 1.5 | 0.9 (2.9)  *(-0.35… +2.23)* | 3.0** | 1.5 (2.3)  *(+0.46… 2.52)* | 0.7 | 0.4 (2.5)  *(-0.74… +1.51)* |
| *Intensity change*  *(-6 dB)* | 1.4 | 0.7 (2.4)  *(-0.35… +1.81)* | 1.6 | 0.7 (2.0)  *(-0.19… +1.54)* | 2.2* | 1.1 (2.4)  *(+0.03… 2.14)* | 1.3 | 0.6 (2.1)  *(-0.32… +1.51)* |
| *Frequency change*  *(+25.5 Hz)* | 2.4* | 1.4 (2.7)  *(+0.19… 2.59)* | 2.2* | 1.3 (2.8)  *(+0.07… 2.56)* | 2.5* | 1.4 (2.5)  *(+0.25… 2.46)* | 2.5* | 1.5 (2.8)  *(+0.21… 2.68)* |
| *Frequency change*  *(-25.5 Hz)* | 1.0 | 0.7 (3.0)  *(-0.66… +1.98)* | 1.4 | 0.9 (2.9)  *(-0.43… +2.17)* | 0.6 | 0.5 (3.7)  *(-1.13… +2.12)* | 0.8 | 0.6 (3.2)  *(-0.85… +1.97)* |
| Control group  LATENCY WINDOW  200–300 MS |  |  |  |  |  |  |  |  |
| *Happy* | 3.9*** | 4.8 (5.3)  *(+2.20… 7.47)* | 5.1*** | 4.7 (3.9)  *(+2.75… 6.63)* | 2.5* | 3.3 (5.6)  *(+0.49… 6.01)* | 3.7** | 3.2 (3.6)  *(+1.33… 4.96)* |
| *Sad* | 1.8 | 2.0 (4.8)  *(-0.34… +4.39)* | 1.8 | 2.1 (5.1)  *(-0.41… +4.62)* | 1.7 | 1.8 (4.5)  *(-0.45… +4.02)* | 2.0 | 2.1 (4.4)  *(-0.08… +4.30)* |
| *Angry* | 2.2* | 1.9 (3.7)  *(+0.11… 3.74)* | 3.0** | 2.3 (3.1)  *(+0.70… 3.80)* | 1.9 | 1.8 (4.1)  *(-0.20… +3.89)* | 1.8 | 1.7 (3.9)  *(-0.26… +3.57)* |
| LATENCY WINDOW  400–500 MS |  |  |  |  |  |  |  |  |
| Emotional stimulus |  |  |  |  |  |  |  |  |
| *Happy* | 3.0** | 4.0 (5.8)  *(+1.18…6.91)* | 3.1** | 3.7 (5.0)  *(+1.17… 6.13)* | 1.5 | 2.5 (6.9)  *(-0.94… +5.95)* | 1.7 | 1.8 (4.6)  *(-0.48… +4.10)* |
| *Sad* | 1.8 | 2.3 (5.4)  *(-0.42… +4.93)* | 1.7 | 2.3 (5.7)  *(-0.55… +5.09)* | 2.0 | 2.0 (4.2)  *(-0.07… +4.09)* | 1.6 | 2.2 (5.8)  *(-0.69… +5.07)* |
| *Angry* | 3.3** | 3.9 (5.1)  *(+1.39… 6.47)* | 3.7** | 3.9 (4.4)  *(+1.67… 6.07)* | 3.7** | 3.5 (4.1)  *(+1.51… 5.57)* | 2.0 | 1.9 (3.9)  *(-0.06… +3.77)* |
| Deviant stimulus |  |  |  |  |  |  |  |  |
| *Vowel duration* | 0.9 | 0.5 (2.4)  *(-0.65… +1.72)* | 1.3 | 0.7 (2.3)  *(-0.40… +1.83)* | 0.8 | 0.5 (2.5)  *(-0.80… +1.72)* | 0.9 | 0.5 (2.4)  *(-0.72… +1.69)* |
| *Vowel change* | 2.7* | 1.0 (1.5)  *(+0.22… 1.68)* | 2.4* | 1.1 (1.9)  *(+0.11… 2.01)* | 0.8 | 0.4 (2.0)  *(-0.61… +1.39)* | 0.8 | 0.4 (2.4)  *(-0.74… +1.64)* |
| *Intensity change*  *(+6 dB)* | 1.8 | 1.2 (2.8)  *(-0.23… +2.56)* | 3.2** | 1.7 (2.2)  *(+0.59… 2.80)* | 1.4 | 1.0 (2.9)  *(-0.48… +2.40)* | 1.0 | 0.6 (2.5)  *(-0.64… +1.83)* |
| *Intensity change*  *(-6 dB)* | 0.2 | 0.2 (3.8)  *(-1.71… +2.08)* | 0.5 | 0.5 (4.2)  *(-1.56… +2.60)* | -0.5 | -0.4 (3.6)  *(-2.18… +1.35)* | -0.4 | -0.03 (3.5)  *(-1.77… +1.71)* |
| *Frequency change*  *(+25.5 Hz)* | 0.6 | 0.5 (3.3)  *(-1.17… +2.14)* | 1.1 | 0.7 (2.8)  *(-0.68… +2.10)* | -1.1 | -0.8 (3.3)  *(-2.49… +0.80)* | -1.0 | -0.6 (2.5)  *(-1.81… +0.63)* |
| *Frequency change*  *(-25.5 Hz)* | 1.2 | 0.7 (2.5)  *(-0.49… +1.94)* | 1.2 | 0.9 (3.0)  *(-0.65… +2.36)* | -0.6 | -0.3 (2.5)  *(-1.59… +0.91)* | 2.1* | 0.9 (1.8)  *(+0.00… 1.80)* |
| LATENCY WINDOW  550–650 MS |  |  |  |  |  |  |  |  |
| Emotional stimulus |  |  |  |  |  |  |  |  |
| *Happy* | 1.5 | 1.8 (5.3)  *(-0.81… +4.41)* | 1.3 | 1.4 (4.4)  *(-0.80… +3.61)* | 0.6 | 0.8 (6.0)  *(-2.17… +3.80)* | 0.3 | 0.4 (4.8)  *(-2.00… +2.72)* |
| *Sad* | 2.4* | 2.9 (5.1)  *(+0.35… 5.38)* | 2.5* | 2.9 (5.0)  *(+0.44… 5.40)* | 2.3* | 2.1 (4.0)  *(+0.16… 4.09)* | 2.0 | 2.4 (5.1)  *(-0.09… +4.94)* |
| *Angry* | 3.1** | 3.6 (5.0)  *(+1.11… 6.06)* | 2.7* | 3.2 (5.1)  *(+0.65… 5.72)* | 2.6* | 3.0 (4.9)  *(+0.52… 5.39)* | 1.6 | 1.5 (3.9)  *(-0.47… +3.42)* |
| Deviant stimulus |  |  |  |  |  |  |  |  |
| *Vowel duration* | 2.0 | 1.5 (3.0)  *(-0.02… +2.93)* | 2.2* | 1.6 (3.2)  *(+0.05… 3.21)* | 0.9 | 0.6 (2.7)  *(-0.76… +1.93)* | 0.8 | 0.6 (3.3)  *(-1.06… +2.25)* |
| *Vowel change* | 3.8*** | 1.4 (1.6)  *(+0.62… 2.21)* | 3.1** | 1.7 (2.3)  *(+0.54… 2.80)* | 0.6 | 0.3 (2.0)  *(-0.72… +1.30)* | 0.8 | 0.5 (2.5)  *(-0.80… +1.72)* |
| *Intensity change*  *(+6 dB)* | 1.7 | 1.4 (3.4)  *(-0.30… +3.07)* | 2.5* | 1.7 (2.9)  *(+0.29… 3.17)* | 1.5 | 1.0 (2.8)  *(-0.41… +2.37)* | 1.1 | 0.7 (2.5)  *(-0.57… +1.91)* |
| *Intensity change*  *(-6 dB)* | 0.4 | 0.3 (3.3)  *(-1.30… +1.95)* | 0.8 | 0.6 (3.5)  *(-1.09… +2.37)* | -0.6 | -0.4 (3.4)  *(-2.11… +1.22)* | 0.5 | 0.3 (3.0)  *(-1.12… +1.78)* |
| *Frequency change*  *(+25.5 Hz)* | 1.3 | 1.1 (3.7)  *(-0.70… +2.93)* | 2.0 | 1.4 (3.0)  *(-0.10… +2.85)* | -0.7 | -0.6 (3.6)  *(-2.39… +1.15)* | -0.33 | -0.2 (2.7)  *(-1.57… +1.14)* |
| *Frequency change*  *(-25.5 Hz)* | 1.5 | 0.9 (2.6)  *(-0.40… +2.18)* | 2.0 | 1.3 (2.8)  *(-0.05… +2.71)* | -0.4 | -0.2 (2.4)  *(-1.40… +0.94)* | 2.5* | 1.3 (2.2)  *(+0.18… 2.33)* |

* *P* < 0.05, ** *P* < 0.01, *** *P* < 0.001

b) Oddball paradigm

|  |  |  | t sig | MEAN (SD)  *(CI)* |  |  |  |  |
| --- | --- | --- | --- | --- | --- | --- | --- | --- |
| Electrode | F3 |  | F4 |  | C3 |  | C4 |  |
| Singing intervention group  LATENCY WINDOW  200–300 MS |  |  |  |  |  |  |  |  |
| *Deviant* | 3.5** | 2.6 (3.5)  *(+1.04… 4.12)* | 3.8*** | 2.7 (3.3)  *(+1.20… 4.10)* | 2.7* | 2.1 (3.6)  *(+0.50… 3.73)* | 2.1* | 1.4 (3.1)  *(+0.02… 2.74)* |
| LATENCY WINDOW  400–500 MS |  |  |  |  |  |  |  |  |
| *Deviant* | 3.2** | 2.6 (3.8)  *(+0.94… 4.31)* | 3.4** | 2.5 (3.5)  *(+0.95… 4.02)* | 2.5* | 2.0 (3.8)  *(+0.35… 3.73)* | 1.9 | 1.3 (3.2)  *(-0.09… +2.72)* |
| LATENCY WINDOW  550–650 MS |  |  |  |  |  |  |  |  |
| *Deviant* | 2.6* | 1.4 (2.6)  *(+0.27… 2.55)* | 2.6* | 1.4 (2.5)  *(+0.29… 2.47)* | 1.2 | 0.7 (2.9)  *(-0.57… +2.00)* | 1.5 | 0.7 (2.1)  *(-0.24… +1.60)* |
| Control group  LATENCY WINDOW  200–300 MS |  |  |  |  |  |  |  |  |
| *Deviant* | 4.4*** | 2.8 (2.7)  *(+1.44… 4.12)* | 3.3** | 2.2 (2.9)  *(+0.78… 3.63)* | 2.7* | 2.4 (3.8)  *(+0.50… 4.26)* | 0.8 | 0.7 (3.8)  *(-1.18… +2.57)* |
| LATENCY WINDOW  400–500 MS |  |  |  |  |  |  |  |  |
| *Deviant* | 4.8*** | 3.5 (3.1)  *(+1.96… 5.05)* | 4.1*** | 3.1 (3.2)  *(+1.52… 4.70)* | 2.8* | 2.7 (4.2)  *(+0.67… 4.80)* | 1.4 | 1.3 (4.2)  *(-0.75… +3.44)* |
| LATENCY WINDOW  550–650 MS |  |  |  |  |  |  |  |  |
| *Deviant* | 4.4*** | 2.9 (2.8)  *(+1.49… 4.24)* | 3.4** | 2.3 (2.9)  *(+0.89… 3.73)* | 2.5* | 2.0 (3.3)  *(+0.32… 3.64)* | 0.9 | 0.8 (3.7)  *(-1.07… +2.63)* |

* *P* < 0.05, ** *P* < 0.01, *** *P* < 0.001

In the latency windows 400–500 ms, rmANOVA showed interaction effect for Stimulus and Electrode [F(24, 13) = 2.998, *p* = 0.001, η^2^ = 0.077], due to responses to different stimuli differing between the four electrodes. Similarly, in the late latency window 550–650 ms, the responses to different stimuli differed significantly between the electrodes and, therefore, an interaction effect of Stimulus and Electrode [F(24, 13) = 2.365, *p* = 0.009, η^2^ = 0.062] was discovered. The detailed *p*-values are reported in Supplementary Table 4.

**Supplementary Table 4.** The detailed rmANOVA *p*-values of the interaction effect of Stimulus and Electrode in the multi-feature paradigm in the latency windows 400–500 ms and 550–650 ms; there were statistical differences between some of the stimuli and electrodes (*p = < 0.05*).

| Stimulus | Electrode (mean) | Electrode (mean) | P-value |
| --- | --- | --- | --- |
| LATENCY WINDOW 400–500 MS |  |  |  |
| *Happy* | C4 (3.730 μV) | F3 (5.866 μV) | *0.014* |
|  |  | F4 (5.828 μV) | *0.004* |
| *Angry* | C4 (3.955 μV) | F3 (6.302 μV) | *0.006* |
|  |  | F4 (6.311 μV) | *0.001* |
|  |  | C3 (5.745 μV) | *0.034* |
| LATENCY WINDOW 550–650 MS |  |  |  |
| *Vowel change* | F4 (2.028 μV) | C3 (0.700 μV)  C4 (1.115 μV) | *0.009*  *0.049* |
| *Sad* | F4 (5.044 μV) | C4 (3.661 μV) | *0.035* |
| *Angy* | C4 (3.134 μV) | F3 (5.250 μV)  F4 (4.882 μV)  C3 (4.809 μV) | *0.008*  *0.024*  *0.045* |

**ITT-analysis**

The rmANOVA was rerun according to the ITT principle so that all available data were included in the analysis (singing intervention group, n = 24; control group, n = 19). Thus, data from two infants in the singing intervention group and one infant in the control group whose parents did not carry out the intervention were added to the filtered data (Supplementary Table 5). The results of the rmANOVA showed the same main and interaction effects and, thus, did not notably differ from the analysis done for the filtered set.

***Latency window 200–300 ms***

The rmANOVA with the added participants showed similarly a main effect of Group [F(1, 39) = 7.729, *p* = 0.008, η^2^ = 0.165], as the singing group infants’ MMRs were larger than those of control group infants (singing group, 5.574 μV; control group, 2.853 μV). A main effect of Stimulus [F(2, 38) = 8.355, *p* = 0.001, η^2^ = 0.176] was found: the MMRs for emotional stimulus *happy* were statistically larger than responses for emotional stimuli *sad, p* = 0.007, and *angry, p* = 0.002 (*happy,* 5.698 μV; *sad,* 3.643 μV; *angry*, 3.298 μV*)*. An interaction effect of Sex and Group [F(1, 39) = 11.972, *p* = 0.001, η^2^ = 0.235] was found, as the MMRs differed between the female and male infants in the singing intervention group, *p* < 0.001 (females, 8.173 μV; males, 2.974 μV).

***Latency window 400–500 ms***

The rmANOVA revealed a main effect of Group [F(1, 39) = 6.633, *p* = 0.014 , η^2^ = 0.145], as the MMRs of the singing group infants were statistically larger and differed from those of the control group infants’ MMRs (singing group, 3.332 μV; control group, 1.462 μV). A main effect of Stimulus [F(8, 32) = 21.861, *p* = < 0.001, η^2 = 0^.359] was found, when the emotional sounds were statistically different than the phonetic deviants. Interaction effect was found for Sex and Group [F(1, 39) = 10.122, *p* = 0.003, η^2^ = 0.206], as the MMRs differed between the female and male infants in the singing intervention group, *p* = 0.001 (singing intervention female 5.060 μV; singing intervention males 1.605 μV). An interaction effect for Group and Stimulus [F(8, 32) = 3.009, *p* = 0.016, η^2^ = 0.072], resulting from the larger MMRs for the singing group and compared with the control group for the phonetic deviant *frequency change +25 Hz*, *p =* 0.036 (singing group, 1.903 μV; control group, 0.213 μV) , emotional stimulus *happy, p =* 0.016 (singing group, 7.102 μV; control group, 3.078 μV), *sad, p =* 0.039 (singing group, 5.713 μV; control group, 2.624 μV), and *angry, p* = 0.012 (singing group, 7.450 μV; control group, 3.496 μV). The rmANOVA showed an interaction effect for Sex and Stimulus [F(8, 32) = 2.686, *p* = 0.028, η^2^ = 0.064], as a result of the MMRs for emotional stimulus *angry* differing statistically from each other between the female and male infants, *p* = 0.022 (female infants, 7.275 μV; male infants, 3.670 μV).

***Latency window 550–650 ms***

In the late latency window, 550–650 ms, rmANOVA revealed a main effect of Group [F(1, 39) = 5.729, *p*= 0.022, η^2^ = 0.128], as the mean magnitude of the MMRs were larger in the singing intervention group when compared to the control group (singing intervention group, 3.121 μV, control group, 1.437 μV). The MMRs to emotional sounds were statistically larger than to the phonetic deviants, leading to a main effect of Stimulus [F(8, 32) = 17.284, *p* = < 0.001, η^2^ = 0.307]. An interaction effect between Sex and Group [F(1,39) = 7.749, *p* = 0.008, η^2^ = 0.166] was discovered, as the MMRs between the female and male infants in the singing intervention group differed from each other, *p* = 0.011 (singing intervention females, 4.426 μV; singing intervention males, 1.817 μV). An interaction effect between Stimulus and Group [F(8, 32) = 4.269, *p* = 0.002, η^2^ = 0.099] was found, due to MMRs to the emotional sounds being larger in the singing intervention group: *happy, p* = 0.009 (singing intervention group, 5.752 μV, control group, 1.487 μV); *sad, p =* 0.003 (singing intervention group, 6.380 μV, control group, 2.811 μV); and *angry*, *p* = 0.033 (singing intervention group, 6.000 μV, control group, 3.148 μV).

**Supplementary Table 5.** The birth characteristics of those two infants in the singing intervention group and one infant in the control group who participated in the AERP measurement but whose parents did not conduct the intervention. When performing the additional ITT analysis, the AERP data of these infants were included to the filtered set.

|  | Singing intervention group | Control group |
| --- | --- | --- |
| Preterm infants, *n* | 2 | 1 |
| Male | 0 | 1 |
| Gestational weeks at birth | 28.7 (26.7–30.7) | 29.7 |
| Weight (g) | 1325 (1120–1530) | 1590 |
| Height (cm) | 38.3 (36.5–40) | 40 |
| SGA^a^ | 0 | 0 |
| Umbilical cord arterial pH | 7.3 (7.28–7.31) | 7.36 |
| Apgar 5 min^b^ | 7.5 (6–9) | 9 |
| Gestational weeks at AERP measurement | 41.5 (40.9–42.1) | 39.3 |

^a^ Born as small for gestational age, birth weight of less than -2 standard deviations for the age. ^b^ Newborn health assessment on a scale 1–10, assessed 5 min postnatally (Breathing effort, heart rate, muscle tone, reflexes and skin color)

**Supplementary Table 6.**  The detailed rmANOVA *p*-values of the main effect of Stimulus in the multi-feature MMN paradigm; emotional stimuli *happy, sad* and *angry* were statistically larger (*p = < 0.05*) than most of the phonetic deviants in the second (400–500 ms) and the late (550–650 ms) latency windows.

| Stimulus | *Happy* | *Sad* | *Angry* |
| --- | --- | --- | --- |
| LATENCY WINDOW  400–500 MS |  |  |  |
| Vowel duration | *0.002* | *0.025* | *< 0.001* |
| Vowel change | *0.001* | *0.027* | *0.001* |
| Intensity change (+6 dB) | *0.007* | 0.172 | *< 0.001* |
| Intensity change (- 6 dB) | *< 0.001* | *< 0.001* | *< 0.001* |
| Frequency change (+25 Hz) | *< 0.001* | *0.001* | *< 0.001* |
| Frequency change (-25 Hz) | *< 0.001* | *0.013* | *< 0.001* |
| LATENCY WINDOW  550–650 MS |  |  |  |
| Vowel duration | 1.000 | *0.008* | *0.028* |
| Vowel change | 0.155 | *< 0.001* | *0.001* |
| Intensity change (+6 dB) | 0.237 | *< 0.001* | *0.001* |
| Intensity change (- 6 dB) | *0.006* | *< 0.001* | *< 0.001* |
| Frequency change (+25 Hz) | 0.169 | *< 0.001* | *0.001* |
| Frequency change (-25 Hz) | 0.059 | *0.001* | *0.001* |

**Supplementary Table 7.** The detailed rmANCOVA *p*-values of the main effect of Stimulus in the multi-feature paradigm in the latency window 550–650 ms (*p = < 0.05*). The emotional sounds differed from most of the phonetic deviants whilst adjusting for ‘Singing days’ in the singing intervention group.

| Stimulus | *Happy* | *Sad* | *Angry* |
| --- | --- | --- | --- |
| LATENCY WINDOW  550–650 MS |  |  |  |
| Vowel duration | 0.525 | *0.028* | *0.037* |
| Vowel change | *0.012* | *< 0.001* | *0.001* |
| Intensity change (+6 dB) | *0.023* | *0.001* | *0.004* |
| Intensity change (- 6 dB) | *0.004* | *< 0.001* | *< 0.001* |
| Frequency change (+25 Hz) | 0.088 | *0.009* | *0.007* |
| Frequency change (-25 Hz) | *0.011* | *< 0.001* | *0.004* |
